# Evaluating the use of a balance prosthesis during balance perturbations in children and young adults with cochleovestibular dysfunction

**DOI:** 10.1038/s41598-023-36613-3

**Published:** 2023-06-15

**Authors:** Rebecca S. Benjamin, Sharon L. Cushing, Alan W. Blakeman, Jennifer L. Campos, Blake C. Papsin, Karen A. Gordon

**Affiliations:** 1 Institute of Medical Sciences, University of Toronto, Toronto, ON Canada; 2 Archie’s Cochlear Implant Laboratory, Hospital for Sick Children, Toronto, ON Canada; 3 Department of Otolaryngology, Head and Neck Surgery, Hospital for Sick Children, Toronto, ON Canada; 4 Department of Otolaryngology, Head and Neck Surgery, University of Toronto, Toronto, ON Canada; 5 Department of Psychology, University of Toronto, Toronto, ON Canada; 6 KITE, Toronto Rehabilitation Institute, University Health Network, Toronto, ON Canada; 7 Department of Communication Disorders, Hospital for Sick Children, Toronto, ON Canada

**Keywords:** Auditory system, Paediatric research

## Abstract

Study objectives were to: (1) quantify stability in children and young adults using cochlear implants with concurrent cochleovestibular dysfunction (CI-V) during balance perturbations and (2) to assess effects of an auditory head-referencing device (BalanCI) on their stability. The BalanCI provides auditory feedback via cochlear implants to cue posture and potentially avoid falling in children with CI-V. It was hypothesized that children and young adults with CI-V respond with larger movements to floor perturbations than typically-developing peers (controls) and that BalanCI use decreases these movements. Motion in response to treadmill perturbations was captured by markers on the head, torso, and feet in eight CI-V and 15 control participants. Stability (area under the curve of motion displacement) and peak displacement latencies were measured. The CI-V group demonstrated less stability and slower responses than the control group during medium and large backwards perturbations (*p*’s < 0.01). In the CI-V group, BalanCI use improved stability during large backwards perturbations (*p* < 0.001), but worsened stability during large sideways perturbations (*p*’s < 0.001). Children and young adults with CI-V move more to remain upright during perturbations than typically-developing peers. The BalanCI has potential to aid physical/vestibular therapy in children with CIs who have poor balance.

## Introduction

The aim of this study was to assess the stability of children and young adults with cochleovestibular dysfunction who use cochlear implants to hear (CI-V group) during balance perturbations compared to typically-developing children (control group) and to determine whether the use of an auditory balance prosthesis supports balance during these tasks. These questions are important because a large proportion (between 20 and 70%) of children with sensorineural hearing loss experience concurrent vestibular^1–3^ and balance^4–6^ problems and are at an increased risk of falls and balance-related injury^7^. In children with cochlear implants (CIs), instability related to vestibular loss can lead to falls and potential head injury; a prior study found that these children have increased risk of device failure and the need for re-implantation compared to children with CIs who have normal vestibular function^7^, suggesting fall-related impacts to the device. Preventing falls in children with cochleovestibular dysfunction can thus result in improved safety for them and reduced risk to their implanted device.

Interventive balance devices provide promise for improved balance in individuals with vestibular dysfunction. Several balance biofeedback devices have successfully been explored, including those that provide vibrotactile^8–16^ or auditory feedback^17–19^ to reference body position at the head^8, 9^ or trunk^8, 13, 14, 16, 17, 19, 20^ so the user can correct their stance and remain upright. Further, several studies have found improved balance in children with cochleovestibular loss who use CIs to hear when their CI is on compared to off^21–24^ suggesting an effect of CI use on balance, potentially thorough improved access to sound cues and thus spatial hearing^25^. Additionally, while CI use aims to stimulate the auditory branch of the vestibulocochlear nerve, it can also stimulate the vestibular branch of the vestibulocochlear nerve^26, 27^ as well as the facial nerve^28, 29^. These findings led to the development of an investigative balance prosthesis, the BalanCI, that provides auditory head referencing cues through the CIs. The BalanCI attaches to and integrates with the user’s CIs. Previous work has shown that BalanCI use improved balance measured by the BOT-2 and the Modified Clinical Test of Sensory Interaction in Balance, which involves stances performed with limited proprioceptive and visual cues, in children with bilateral cochleovestibular dysfunction and bilateral hearing loss^26^.

One common assessment of balance is the Bruininks-Oseretsky Test of Motor Proficiency Second Edition (BOT-2)^30^. Several studies have shown poorer balance as measured by the BOT-2 in children with unilateral^21, 31–34^ and bilateral^32–34^ cochleovestibular dysfunction than typically-developing children. While the BOT-2 can help identify balance deficits in children, significant effects of balance interventions were limited to only one of the subtasks (tandem stance, eyes closed) because the other tasks were either too easy (producing ceiling effects) or too difficult (producing floor effects) to be altered by the intervention^26^. Moreover, many of the BOT-2 tasks are stationary which may not reflect more dynamic balancing encountered in daily life. It may thus be helpful to utilize other balance measures involving a gradient of difficulty to assess balance deficits as well as to examine the benefits of balance interventions in children with cochleovestibular dysfunction.

Translational balance perturbations mimic a slip or a trip, reflecting a frequently encountered balance situation in which posture must be maintained to prevent a fall. Translational balance perturbations have commonly been used to test reactive balance in older adults^35–37^ as well as in patients with conditions associated with gait and balance problems, such as Parkinson’s disease^38–40^ and stroke^41, 42^. In adults with cochleovestibular dysfunction, externally-induced translational balance perturbations resulted in less stability than in healthy adults, as indicated by kinematic measures such as angular head and torso displacement^43, 44^ and poor responses to tilt perturbations were improved by training^45^. By utilizing balance perturbations, we can stimulate potential falls that could otherwise result in bodily harm and risk CI failure in this clinical population, and evaluate whether an auditory balance prothesis might help reduce these risks by improving stability. To our knowledge, the present study is the first to examine externally-induced balance perturbations in children with cochleovestibular dysfunction who use CIs.

The present study tested the hypotheses that children with cochleovestibular dysfunction who use bilateral CIs show impaired stability during balance perturbations relative to typically developing peers and that stability and response latency during balance perturbations improves with BalanCI use in the CI-V group.

## Methods

Approval for this study was obtained from Health Canada (Investigational Testing Authorization, Class II, #267523), The Hospital for Sick Children’s Research Ethics Board (#1000057107) and the University Health Network’s Research Ethics Board (#17-5896.3), Medical Engineering (#267523) and Tissue, Data, and Medical Device Committee. All research was performed in accordance with relevant guidelines and regulations. Informed written consent was obtained from participants. For children who were too young or unable to consent, informed written consent from guardians and verbal assent from participants were obtained.

### Participants

Fifteen typically-developing children (control group: mean ± SD = 13.6 ± 2.75 years of age, 6 female) and eight children and young adults with cochleovestibular dysfunction (CI-V group: 19.5 ± 5.45 years of age, 6 female) participated in this study. Typically-developing participants were recruited from a control participant contact list with the following inclusion criteria: normal hearing, self-reported typical development, and normal balance function on the BOT-2. Of the 15 children in this group, three self-reported normal hearing and twelve exhibited thresholds ≤ 25 dB at 250, 500, 1000, 2000, 4000 and 8000 Hz). Eight participants with bilateral cochleovestibular dysfunction were recruited from the Hospital for Sick Children’s Cochlear Implant Program. Inclusion criteria for these participants were: bilateral deafness onset in childhood (< 18 years of age), clinical findings of bilateral vestibular dysfunction and bilateral cochlear implantation in childhood (< 18 years of age). All of these participants had abnormal bilateral findings on at least one test assessing semi-circular canal function and the vestibular ocular reflex (caloric testing, rotational chair, or video head impulse test). Five of eight participants also had abnormal bilateral otolith dysfunction as tested by recording cervical vestibular-evoked myogenic potentials (cVEMPs). Of the three remaining participants, two did not undergo cVEMP testing, and one participant had unilateral abnormal cVEMP results. At the time of testing, ocular vestibular-evoked myogenic potentials were not a part of the clinical vestibular test battery in our program. Exclusionary criteria were inability or unwillingness to perform all measures of the test protocol. Information on demographics and etiology for all participants are detailed in Table 1.

**Table 1:** Demographic and etiological data of participants.

Participant	Age at test (years)	Sex	Etiology of deafness	Age at first implant (years)	Age at second implant (years)
CI group					
1	9	Female	Unknown	1.16	1.16
2	17	Male	Usher’s	2.9	2.9
3	18	Female	Abnormal cochlea	2.04	6.16
4	19	Male	CMV	14.86	16.93
5	20	Female	Meningitis	0.91	7.39
6	21	Female	Meningitis	3.36	8.28
7	25	Female	Meningitis	4.71	16.07
8	27	Female	CMV	15.39	16.88
Typically-developing group					
1	7	Female			
2	10	Female			
3	11	Male			
4	13	Male			
5	13	Male			
6	13	Male			
7	13	Female			
8	14	Male			
9	14	Female			
10	15	Male			
11	15	Male			
12	16	Male			
13	16	Female			
14	16	Female			
15	18	Male			

### Bruininks-Oseretsky Test of Motor Proficiency Second Edition (BOT-2), balance subset

Participants completed the balance subset of the BOT-2 which is an assessment of static and dynamic balance used in children aged 4–21 years^30^. Wearing a five-point harness for safety, participants completed the 9 tasks in the battery. Static tasks were scored by the length of time for which the participant was able to hold the stance with a goal of 10 s, and dynamic tasks were scored by the number of successful steps achieved with a goal of 6 steps. The trial ended if the participant stepped out of position, lifted their hands from their hips, or opened their eyes during an “eyes closed” task. Time to fall/number of steps for each trial was converted into a numeric score^30^. The best score of two attempts was used for each task. The points from each task were summed, and these raw scores were adjusted by age and sex to produce a scaled score out of 35^30^. As we would not expect to see balance ability decrease between the ages of 21 to 26 years^46–48^, scores from participants over the age of 21 years (n = 3) were converted using 21 years as their age. Performance on the BOT-2 was measured by time to fall/number of steps for each task, and overall scaled score.

### BalanCI

Participants in the CI-V group were provided with a pair of laboratory-owned Nucleus 6 CP910 CI speech processors containing their most recent MAPs and typical settings for use during the study. The BalanCI was attached between the speech processor and battery on the right ear, and a cable connected the BalanCI to the accessory port of each processor. For participants in the control group, the BalanCI was attached to a CI speech processor on the right ear and connected to a portable amplifier which input to a pair of insert earphones to deliver the acoustic cues. The earphones were fitted with hearing aid open domes to ensure access to surrounding sounds in the environment. This additional equipment was housed in a small pack worn around the waist.

Before the study began, position of the BalanCI was calibrated for head centered position and checked for function in the head pitched downwards position. The participant was then given time (up to 15 min) to learn how to use the device. They were instructed to move their head slowly back and forth and side to side, and to identify the boundaries where the cues began playing. The BalanCI’s auditory stimuli were activated when head position deviated more than 5° from center in the pitch and roll planes. The frequency of the auditory stimuli was modulated based on the pitch of the head, with the frequency increasing as the head pitched forwards, and decreasing as the head pitched backwards (100 to 780 Hz). The amplitude of the auditory stimuli modulated based on the roll of the head, with a larger amplitude on the side that the head was leaning towards. The volume of the BalanCI was audible and set to a comfortably loud level for each participant based on behavioral feedback.

### Motion capture measures

**Figure 1:**
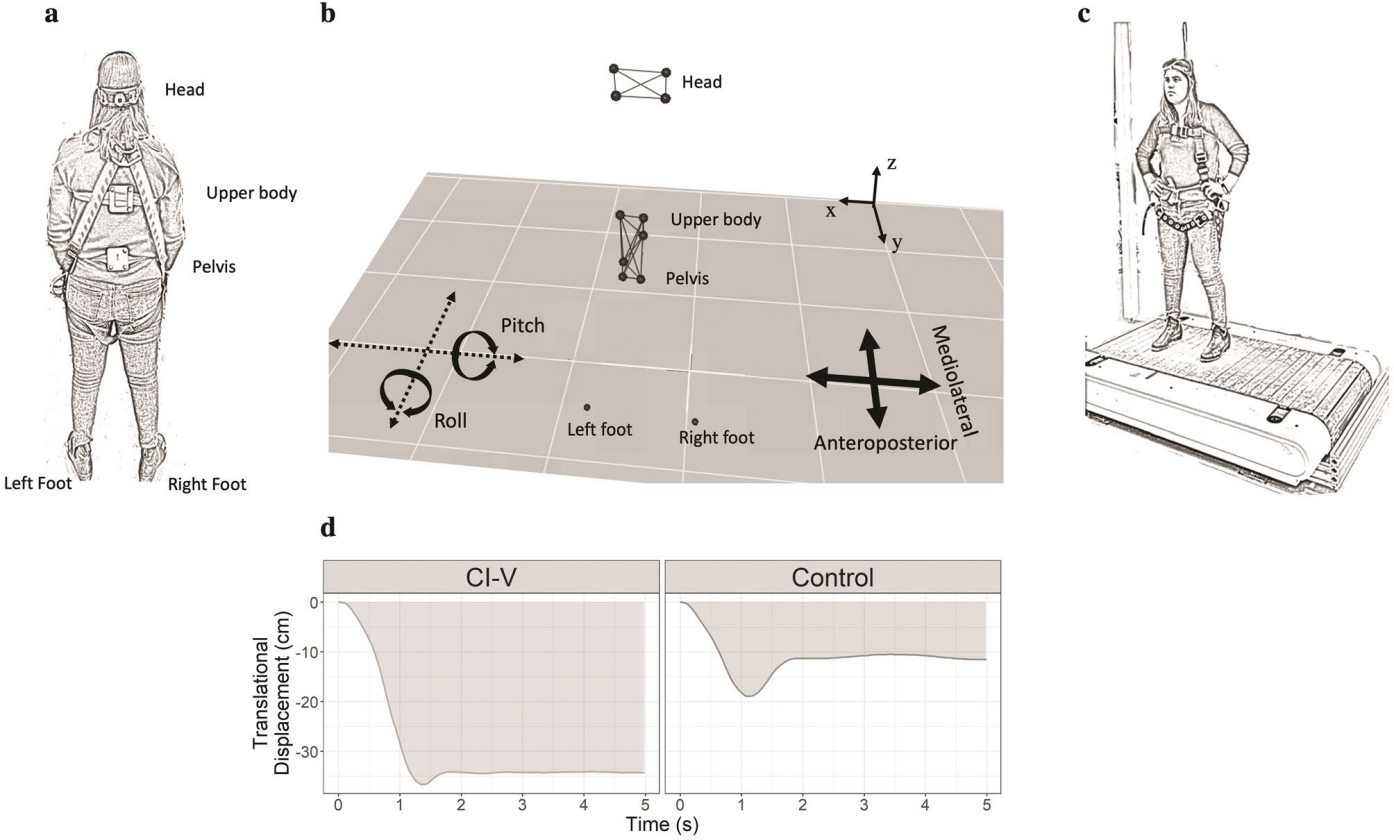
(**a**) Placement of head, upper body, pelvis, and foot markers. (**b**) Representation of markers in space as measured by motion capture software, and assessment of translational (anteroposterior and mediolateral) and rotational (pitch and roll) movements. (**c**) Treadmill set-up with harness. (**d**) Waveforms were created for translational and rotational motion for each marker cluster, and AUC was determined as a measure of overall magnitude and duration of movements. Pictured is an example of AUC during a large backwards perturbation of a pelvis marker in one participant in the CI-V group, and one participant in the control group.

Ten Raptor-E cameras and Cortex (Motion Analysis Corporation, Santa Rosa, CA) were used to collect kinematic data at 250 frames/second. Participants wore 4 reflective markers on the head (arranged on a modified bicycle helmet interior), 3 on the upper body, 3 on the pelvis (both upper body and pelvis clusters were secured to the participant using TheraBands), and one on each foot (taped to the participant’s shoe at the fifth metatarsal) (Fig. 1a and b). A treadmill marker cluster was also used to record treadmill belt motion, consisting of 3 motion capture makers placed in a right angle on a rubber door stopper. Motion capture data was post-processed in Cortex. Large marker dropouts were excluded from the study. Recordings were exported as C3D files for analysis in MATLAB. For body segments containing more than one marker (head, upper body and pelvis), translational motion was averaged over the markers within the cluster to produce the average translation for the body segment. .csv files containing average translational and rotational motion (shown in Fig. 1b) at 0.02 s intervals for each marker cluster were produced using MATLAB. As 3 markers are required within a body segment to calculate rotation, rotational data was not produced for foot markers. As shown in Fig. 1c, participants faced the direction of motion and were secured by a harness.

### Perturbation task stimuli

Small, medium, and large perturbations were delivered in the forwards, backwards, left and right directions by a treadmill (Woodway PPS70) (Fig. 1c). The velocities of the perturbations (Table 2) were selected through piloting and the limits of the treadmill and are consistent with other balance perturbation studies^49–51^. Treadmill perturbation displacement was recorded using a treadmill marker cluster placed on the treadmill belt. The order of treadmill perturbation stimuli was randomized. While participants were cued to the direction of the perturbation by their position on the treadmill, the magnitude of the perturbation was randomized and the onset was jittered (0 to 4 s) to reduce anticipatory effects. 24 trials were completed per block (4 directions × 3 magnitudes × 2 trials each). One block was completed with the BalanCI off, and one was completed with the BalanCI on. The order of blocks was randomized. Participants were given the opportunity to observe a medium treadmill perturbation before beginning the task to ensure they felt comfortable completing the task.

**Table 2:** Treadmill perturbation stimuli.

Perturbation direction	Mean small velocity ± SD (cm/s)	Mean medium velocity ± SD (cm/s)	Mean large velocity ± SD (cm/s)
Forwards	9.87 ± 1.08	19.79 ± 1.37	27.98 ± 1.76
Backwards	9.53 ± 0.87	19.94 ± 1.30	27.86 ± 1.47
Left	9.98 ± 1.88	20.01 ± 1.26	28.64 ± 1.75
Right	10.00 ± 2.15	19.95 ± 1.31	28.09 ± 1.75

### Perturbation task procedure

As shown in Fig. 1c, participants wore a harness for safety while on the treadmill. As the treadmill was only able to move in one direction, participants faced toward the front or back of the treadmill for forward and backward perturbations, respectively, and were turned 90 degrees to the left or right for left and right sideways perturbations, respectively. They were instructed to remain still and upright in response to perturbations and were permitted to step to prevent a fall. At the start of each trial, they were instructed to stand with their feet shoulder-width apart on the “start” line, marked on the treadmill with tape, with their hands on their hips. The treadmill motion capture cluster was also placed on the “start” line on the treadmill belt. Once the participant indicated they were ready for the perturbation, the motion capture cameras started collecting data. The perturbation onset was randomly jittered (0 to 4 s) from this time point. Participants were told to hold their final position in order to determine completion of each trial. The participant was then instructed to return to the “start” line on the treadmill in the direction of the next perturbation trial. Each participant completed 2 trials in 4 directions and 3 magnitudes presented in random order.

### Perturbation task measures

Waveforms were produced for translational and rotational displacement over time for each marker cluster during each perturbation. Stability was quantified as the area under the curve (AUC) of these waveforms for displacement (Fig. 1d) in the same axis and plane as treadmill motion for translational (anterior–posterior axis for forwards/backwards perturbations, and medio-lateral for sideways perturbations) and rotational (pitch for forwards/backwards perturbations, and roll for sideways perturbations) motion, respectively (Fig. 1b). Peaks of motion waveforms were also analyzed to better understand the temporal qualities of responses. The period of interest of all perturbations was from 0 to 4 s after treadmill motion onset. For all translational measures, the displacement of the treadmill marker cluster was subtracted from the displacement of the body segment clusters.

### Analyses

Data analyses were completed in R-Studio (4.0.4). For each perturbation trial, absolute AUC was produced using the AUC function and trapezoid method (DescTools package). This measure was selected to provide an overall indication of degree and duration of movement. In addition to AUC, the first and second peak in translational and rotational displacement were selected for each trial. The first peak (P1) was selected as the first absolute peak between 0 and 1.5 s after treadmill onset, and the second peak (P2) was the next absolute peak in the opposite direction between P1 and 2 s after treadmill onset. For each peak, latency was determined. Inter-peak latency (the absolute amount of time between P1 and P2) was also determined to further characterize the overall time course of perturbation responses.

A Welch two sample *t* test was conducted to examine scaled total BOT-2 scores between groups. BOT-2 performance by task and perturbation data were analyzed in R using linear mixed models (lmer function, lme4 package). Mixed linear models were chosen as they account for both fixed and random factors and are robust measures to predict outcome variability^52^. The model used for the BOT-2 data was: “value ~ Group*`Task Number` + Sex + Age + (1|`Study ID`)”. Age and sex were included as fixed effects in the model to predict BOT-2. A consistent model was used to assess each type of perturbation data (translational and rotational displacement in each direction: AUC, P1 latency, P2 latency, inter-peak latency): “value ~ BalanCI*Perturbation Size*Group*Marker + Age + Trial order + Sex + Height + (1|`Study ID`)”. Age, sex, relative height (measured as vertical distance between the marker on the left back of the head and the left foot marker) and trial number were included as fixed effects for models predicting perturbation data. Individual participants were included as a random effect in all models. ANOVAs were conducted using Satterthwaite’s method on each model, and interactions of interest were investigated using pairwise comparisons and were adjusted for multiple comparisons using the Kenward-Roger method. Motion capture findings were averaged over all marker clusters to focus on the aims of the present study (effects of group and BalanCI on stability). Assumptions of mixed-model assumptions were assessed. Some models revealed deviations likely due to marker differences. These were not further explored as they were outside the scope of the present paper. Each model and results of associated ANOVAs are shown in supplementary data (Supplementary data Tables 1–14). Data used in analyses can be made available upon request.

## Results

One of the 15 typically-developing children in the control group did not complete the “BalanCI on” conditions of the perturbation task due to equipment difficulties. All other participants completed all components of the study. One of the 8 participants in the CI-V group lost their balance during perturbation testing, requiring the support of the harness during only one trial. All other participants in both groups were able to remain upright during all treadmill perturbation trials.

The CI-V group was significantly older than the control group (mean age difference = 5.9 years, *t*(21) = 3.49, *p* = 0.002, *Cohen’s d* = 1.53, 95% CI [2.38 years, 9.42 years], two-sided). There was no significant difference in relative height, *t*(20) = 0.91, *p* = 0.38, (two-sided), or sex, (*p* = 0.19, Fisher’s exact test) between groups.

All main effects and interactions are shown in the supplementary data (Supplementary data, Tables 1–14) and the contrasts particular to the study objectives are detailed below.

### Static and dynamic balance was poor in the CI-V group

**Figure 2:**
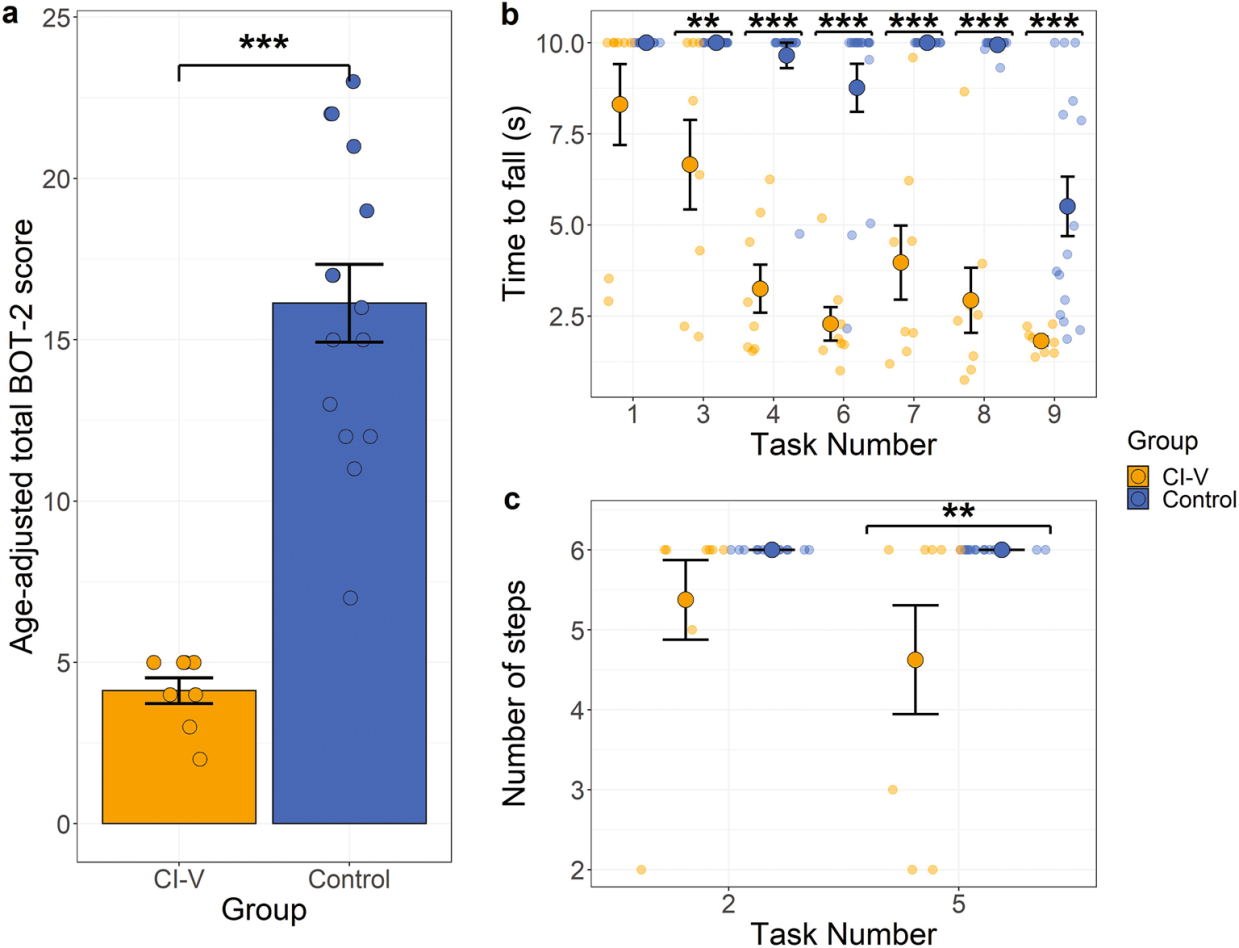
(**a**) Overall performance on the BOT-2 by group on the x-axis, and age- and sex-scaled total BOT-2 scores on the y-axis. (**b**) Performance on BOT-2 static tasks with task number on the x-axis, and time to fall (s) on the y-axis. The static tasks are (1) standing in tandem stance on a stable surface with eyes open, (3) standing on one foot on a stable surface with eyes open, (4) standing in tandem stance on a stable surface with eyes closed, (6) standing on one foot on a stable surface with eyes closed, (7) standing on one foot on a balance beam with eyes open, (8) standing heel-to-toe on a balance beam with eyes open, and (9) standing on one foot on a balance beam with eyes closed. (**c**) Performance on the BOT-2 dynamic tasks with task number on the x-axis, and number of successful steps achieved on the y-axis. The dynamic tasks are (2) walking forward on a line, and (5) walking heel-to-toe on a line. The CI-V group (n = 8) is indicated in orange, and control group (n = 15) in blue. Error bars represent standard error of the mean, and points represent individual datapoints. Significance differences are indicated: **p < 0.01, ***p < 0.001.

As shown in Fig. 2a, the CI-V group performed more poorly on the BOT-2 overall than typically developing children in the control group (mean scaled-score group difference = 12 points, *t*(16.82) = − 9.45, *p* < 0.0001, *Cohen’s d* = − 3.10, 95% CI [− 14.69 points, − 9.33 points], (two-sided). The CI-V group had particular difficulties on some of the static tasks (Fig. 2b): standing on one foot with eyes open (mean group difference = 3.34 s, *p* = 0.002, *Cohen’s d* = − 2.24, 95% CI [0.89 s, − 7.17 s]), standing with eyes closed in a tandem stance (mean group difference = 6.4 s, *p* < 0.0001, *Cohen’s d* = − 3.94, 95% CI’s [3.94 s, 10.23 s]), and standing with eyes closed on one foot (mean group difference = 6.47 s, p < 0.0001, *Cohen’s d* = − 3.98, 95% CI [4.02 s, 10.3 s]) compared to the control group. Balance beam tasks were also challenging for the CI-V group: standing on one foot (mean group difference = 6.03 s, *p* < 0.0001, *Cohen’s d* = − 3.74, 95% CI [3.59 s, 9.86 s]) and heel-to-toe (mean group difference = 7.01 s, *p* = 0.0005, *Cohen’s d* = − 4.28, 95% CI [4.55 s, 10.84 s]) with eyes open, and one foot with eyes closed (mean group difference = 3.69 s,* p* < 0.001, *Cohen’s d* = − 2.44, 95% CI [1.24 s, 7.52 s]). As shown in Fig. 2c, the CI-V group also showed fewer steps walking heel-to-toe on a line than participants in the control group (mean group difference = 1.37 steps, *p* = 0.009, *Cohen’s d* = − 3.28, 95% CI [0.4 steps, 3.32 steps]).

### Group differences in overall movement in response to perturbations

**Figure 3:**
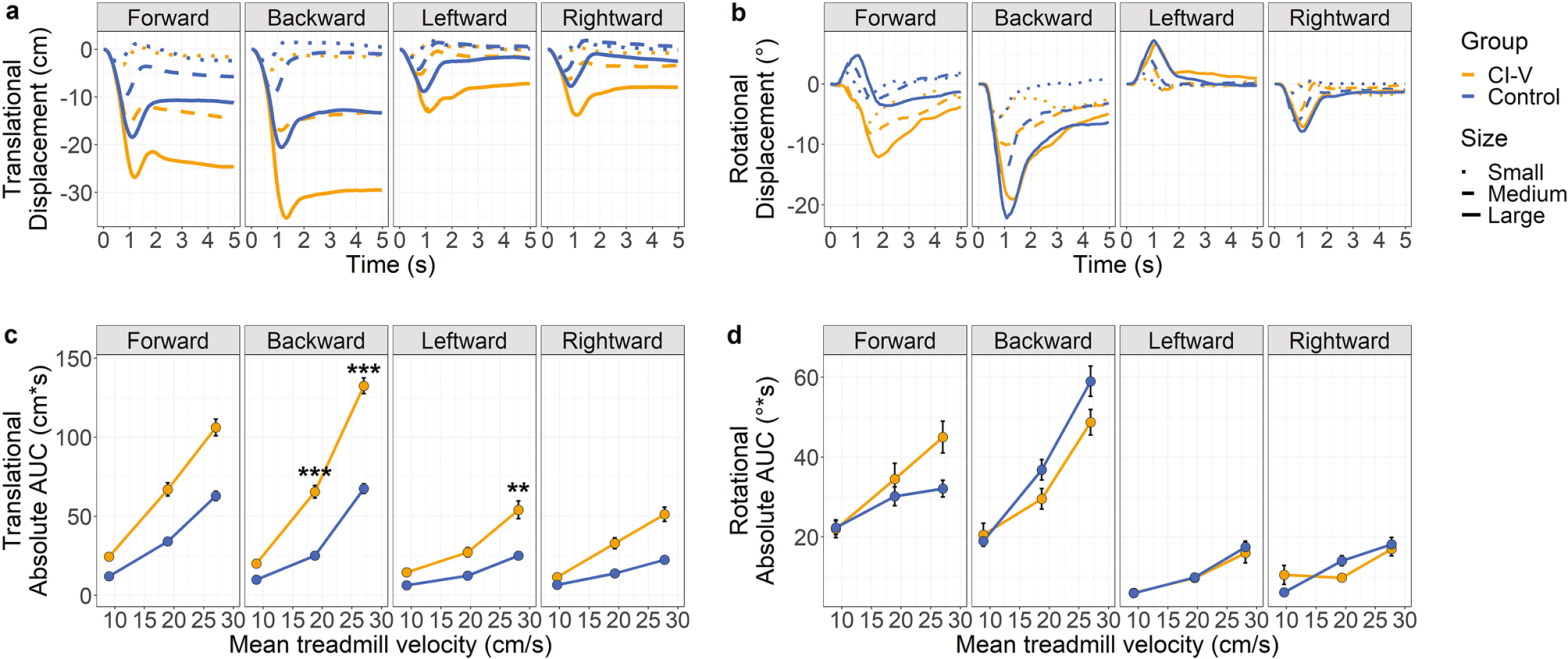
(**a**) Translational displacement (cm) over time (s) and (**b**) rotational displacement (°) over time (s) collapsed over all motion capture clusters and BalanCI conditions for small, medium, and large treadmill perturbations in the forwards, backwards, leftward, and rightward directions. Anteroposterior axis and pitch plane are indicated for forwards and backwards perturbations, and mediolateral axis and roll plane are indicated for leftwards and rightwards perturbations. Translational motion: positive values are in the same direction as treadmill motion. Rotational motion: positive is pitch upwards and roll rightwards. (**c**) Absolute AUC for translational motion (cm*s) and (**d**) rotational motion (°*s) across small, medium, and large treadmill perturbations, plotted as mean treadmill velocity (cm/s) and collapsed across marker and BalanCI condition. The CI-V group (n = 8) is in orange, and control group (n = 15) in blue. Error bars represent standard error of the mean, and points represent means. Significance differences are indicated: **p < 0.01, ***p < 0.001.

Figure 3 plots the grand-average translational motion (a) and rotational motion (b) over trial time across participants and markers in each group in response to small, medium, and large perturbations from four directions (forward, backward, left, and right). Data across trials with and without the BalanCI are shown. As plotted in Fig. 3c, translational movement, measured as AUC, significantly increased with perturbation size across all four directions (forwards: *F*(2, 1248.25) = 334.52, *p* < 0.0001, *η*_p_^2^ = 0.35; backwards: *F*(2, 1263.72) = 595.42, *p* < 0.0001, *η*_p_^2^ = 0.49; left: *F*(2, 1272.48) = 126.03, *p* < 0.0001, *η*_p_^2^ = 0.17; and right: *F*(2, 1283.41) = 147.08, *p* < 0.0001, *η*_p_^2^ = 0.19). Participants in the CI-V group showed increased translational movement compared to controls for medium (mean group difference = 40.39 cm*s, *p* = 0.0002, *Cohen’s d* = − 1.39, 95% CI [20.74 cm*s, 78.6 cm*s]) and large (mean group difference = 64.96 cm*s, *p* < 0.0001, *Cohen’s d* = − 2.06, 95% CI [44.41 cm*s, 102.9 cm*s]) backwards perturbations, and large leftward perturbations (mean group difference = 28.93 cm*s, *p* = 0.01, *Cohen’s d* = − 1.45, 95% CI [6.79 cm*s, 70.2 cm*s]). Data shown in Fig. 3d revealed that rotational movement (AUC) also increased with perturbation size (forwards: *F*(2, 711.05) = 28.8, *p* < 0.0001, *η*_p_^2^ = 0.07; backwards: F(2, 739.76) = 95.54, *p* < 0.0001, *η*_p_^2^ = 0.21; left: F(2, 717.22) = 52.72, *p* < 0.0001, *η*_p_^2^ = 0.13; and right: *F*(2, 757.6) = 30.02, *p* < 0.0001, *η*_p_^2^ = 0.07, perturbations) but that there were no main group differences in rotational movement (*p* > 0.1). Age had no effect on translational or rotational AUC for any direction (*p*’s ≥ 0.05).

### BalanCI has effects on overall movement in response to large perturbations

**Figure 4:**
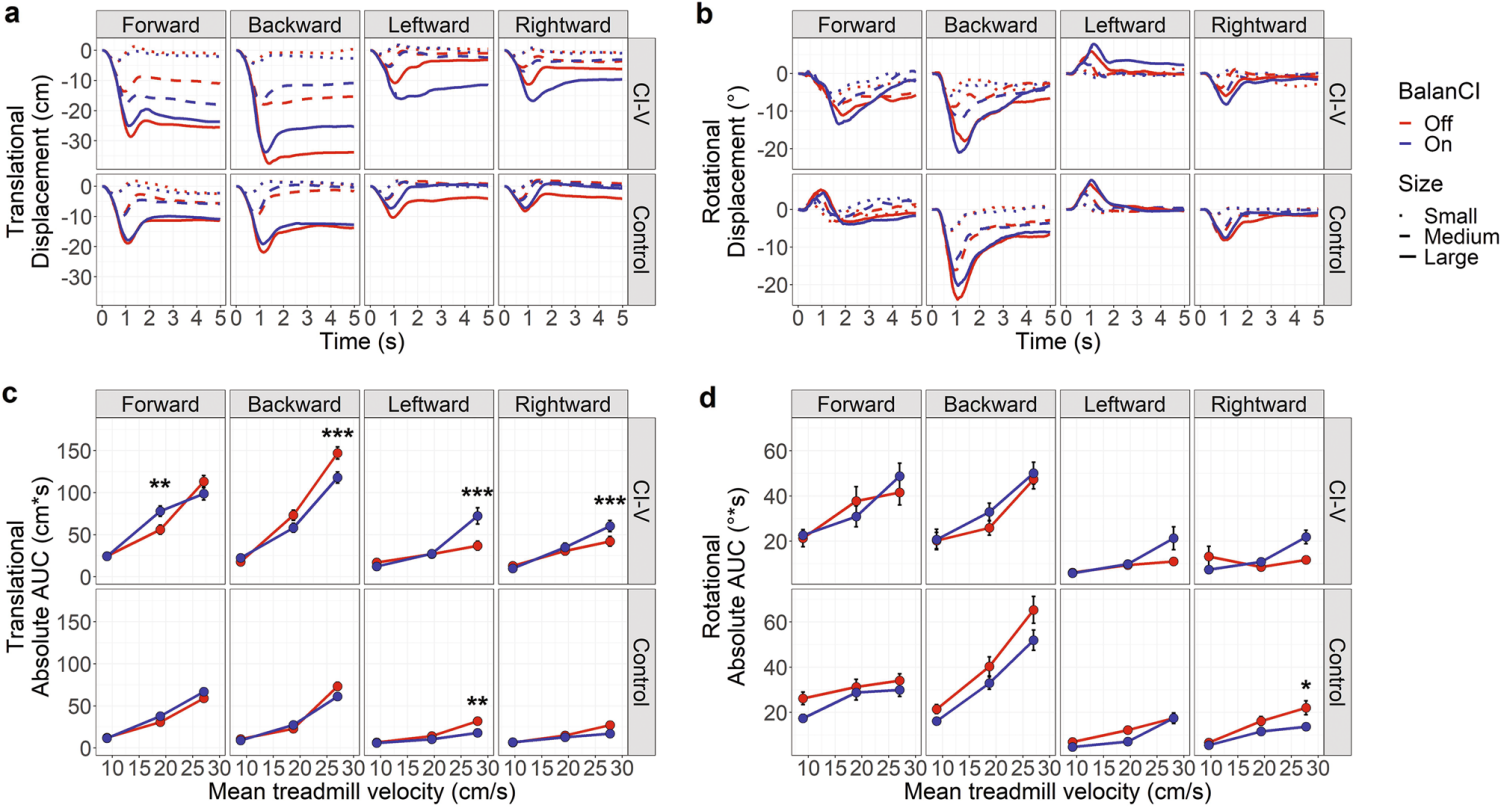
(**a**) Translational displacement (cm) over time (s) and (**b**) rotational displacement (°) over time (s) collapsed over all motion capture clusters for small, medium and large treadmill perturbations in the forwards, backwards, leftward, and rightward directions in the CI-V (n = 8) and control (n = 15) groups, with BalanCI off and on. Anteroposterior axis and pitch plane are indicated for forwards and backwards perturbations, and mediolateral axis and roll plane are indicated for leftwards and rightwards perturbations. Translational motion: positive values are in the same direction as treadmill motion. Rotational motion: positive is pitch upwards and roll rightwards. (**c**) Absolute AUC for translational motion (cm*s) and (**d**) rotational motion (°*s) across small, medium, and large treadmill perturbations, plotted as mean treadmill velocity (cm/s) and collapsed across markers. BalanCI off is in red, and on in blue. Error bars represent standard error of the mean, and points represent means. Significance differences are indicated: *p < 0.05, **p < 0.01, ***p < 0.001.

Figure 4 plots the grand-average translational (a) and rotational motion (b) shown in Fig. 3 separated for conditions in which BalanCI was off versus on. Effects of BalanCI were minimal in the control group for both translational or rotational movements (Fig. 4c and d, respectively); control participants exhibited smaller translational movements (AUC) with the BalanCI on during large left perturbations (mean BalanCI difference = 13.95 cm*s, *p* = 0.002, *Cohen’s d* = 0.50, 95% CI [2.92 cm*s, 23.46 cm*s]), and smaller rotational movements (AUC) with the BalanCI on during large right perturbations (mean BalanCI difference = 8.35°*s, *p* = 0.026, *Cohen’s d* = 0.54, 95% CI [0.47°*s, 14.94°*s]). In the CI-V group, results were mixed: translational movements (AUC) were reduced for large backward perturbations (mean BalanCI difference = 29.16 cm*s, *p* < 0.0001, *Cohen’s d* = 0.83, 95% CI [11.75 cm*s, 47.88 cm*s]) with the BalanCI on but higher for medium forward (mean BalanCI difference = 22 cm*s, *p* = 0.01, *Cohen’s d* = -0.59, 95% CI [2.75 cm*s, 21.84 cm*s]) and large left (mean BalanCI difference = 35.28 cm*s, *p* < 0.0001, *Cohen’s d* = − 1.31, 95% CI [20.86 cm*s, 48.83 cm*s]) and right (mean BalanCI difference = 18.1 cm*s, *p* = 0.0001, *Cohen’s d* = − 0.75, 95% CI [5.75 cm*s, 29.74 cm*s]) perturbations with the BalanCI on. No significant effects of BalanCI were found on rotational movement (AUC) in the CI-V group (*p* > 0.05).

### Group differences in perturbation response latencies

**Figure 5:**
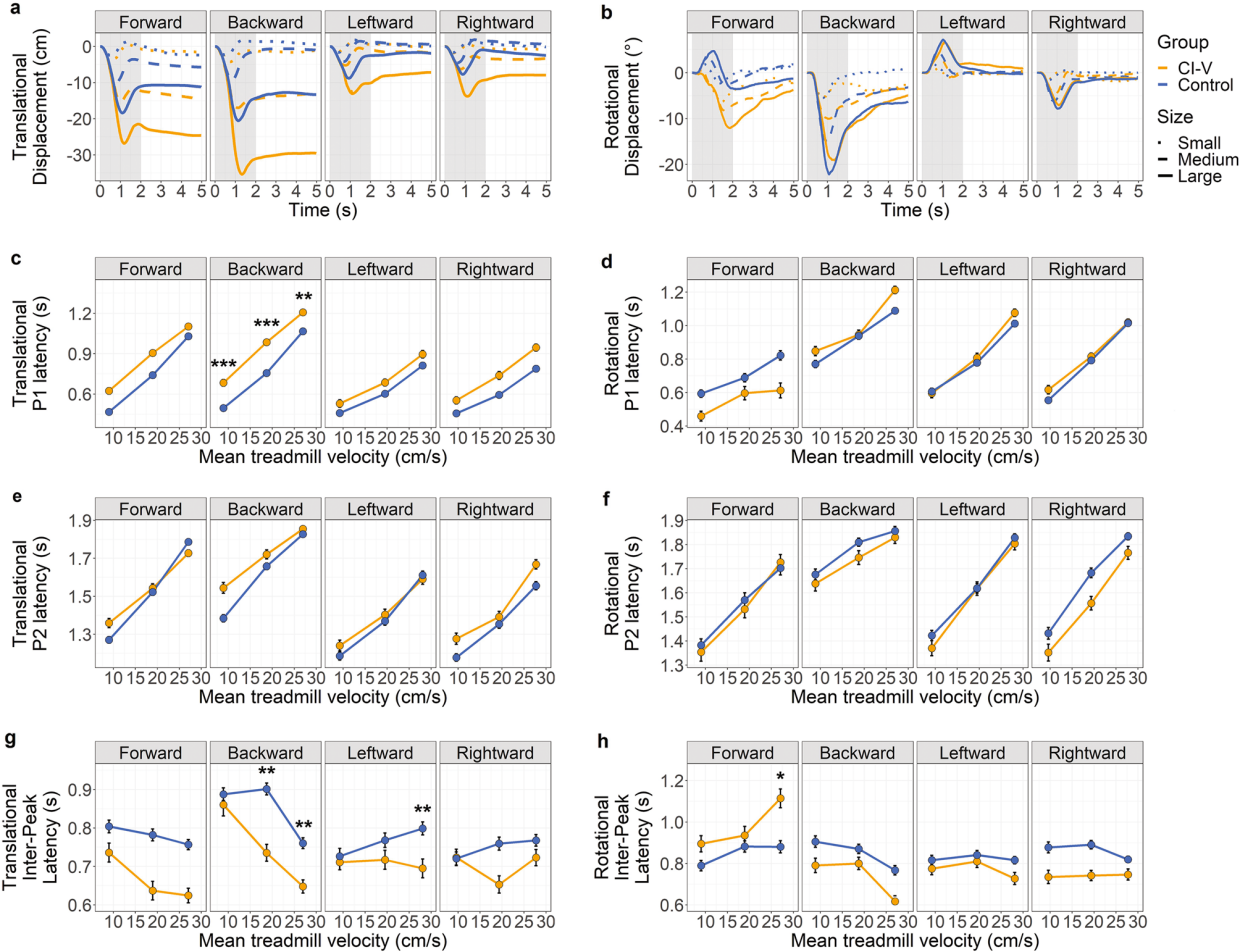
(**a**) Translational displacement (cm) over time (s) and (**b**) rotational displacement (°) over time (s) collapsed over all motion capture clusters and BalanCI conditions for small, medium, and large treadmill perturbations in the forwards, backwards, leftward, and rightward directions. Anteroposterior axis and pitch plane are indicated for forwards and backwards perturbations, and mediolateral axis and roll plane are indicated for leftwards and rightwards perturbations. Translational motion: positive values are in the same direction as treadmill motion. Rotational motion: positive is pitch upwards and roll rightwards. The shaded region represents the region of interest for peak selection. Peak 1 latency (s) for (**c**) translational and (**d**) rotational motion across small, medium, and large treadmill perturbations, plotted as mean treadmill velocity (cm/s). Peak 2 latency (s) for (**e**) translational and (**f**) rotational motion across perturbation degrees. Inter-peak latency (P2 latency – P1 latency) for (**g**) translational and (**h**) rotational motion across perturbation degrees. The CI-V group (n = 8) is in orange, and control group (n = 15) in blue. All measures are collapsed over motion capture clusters. Error bars represent standard error from the mean, and points represent means. Significance differences are indicated: *p < 0.05, **p < 0.01, ***p < 0.001.

Figure 5 plots the grand-average translational motion (a) and rotational motion (b) over trial time across participants and markers in each group during small, medium, and large perturbations for each direction (forward, backward, left and right), with the region of interest for peak selection indicated by the shaded region. Peak latencies were measured to assess delays in movement in response to perturbations. As shown in Fig. 5c, translational P1 latency is significantly delayed with increased perturbation size across all 4 directions (forwards: *F*(2, 1247.77) = 873.1, *p* < 0.0001, *η*_*p*_^*2*^ = 0.58; backwards: *F*(2, 1263.76) = 987.85, *p* < 0.0001, *η*_p_^2^ = 0.61; left: *F*(2, 1273.95) = 216.11, *p* < 0.0001, *η*_p_^2^ = 0.25; and right: *F*(2, 1285.8) = 224.38, *p* < 0.0001, *η*_p_^2^ = 0.26, perturbations). The CI-V group exhibited a later translational P1 than the control group during small (mean group difference = 0.18 s, *p* = 0.0005, *Cohen’s d* = 1.37, 95% CI [0.09 s, 0.39 s]), medium (mean group difference = 0.23 s, *p* = 0.0001, *Cohen’s d* = 1.59, 95% CI [0.13 s, 0.43 s]), and large (mean group difference = 0.14 s, *p* = 0.0061, *Cohen’s d* = 1.09, 95% CI [0.04 s, 0.43 s]) backwards perturbations. As shown in Fig. 5d, rotational P1 latency was also significantly delayed with increased perturbation size (forwards: *F*(2, 736.42) = 26.8, *p* < 0.0001, *η*_p_^2^ = 0.07; backwards: *F*(2, 741.58) = 156.73, *p* < 0.0001, *η*_p_^2^ = 0.3; left: *F*(2, 747.29) = 243.61, *p* < 0.0001, *η*_p_^2^ = 0.39; and right: *F*(2, 756.29) = 375.35, *p* < 0.0001, *η*_p_^2^ = 0.5, perturbations).

P2 latency is plotted in Fig. 5e and f for translational and rotational motion, respectively. For both translational and rotational measures, P2 latency was significantly delayed with increased perturbation size for translational (forwards: *F*(2, 1248.63) = 423.13, *p* < 0.0001, *η*_p_^2^ = 0.4; backwards, *F*(2, 1262.84) = 286.37, *p* < 0.0001, *η*_p_^2^ = 0.31; left: *F*(2, 1273.94) = 181.03, *p* < 0.0001, *η*_p_^2^ = 0.22; and right: *F*(2, 1286.16) = 190.57, *p* < 0.0001, *η*_p_^2^ = 0.23) and rotational (forwards: *F*(2, 736.58) = 65.91, *p* < 0.0001, *η*_p_^2^ = 0.15; backwards: *F*(2, 742.05) = 35.47, *p* < 0.0001, *η*_p_^2^ = 0.09; left: *F*(2, 747.61) = 161.77, *p* < 0.0001, *η*_p_^2^ = 0.3; and right: *F*(2, 756.38) = 155.07, *p* < 0.0001, *η*_p_^2^ = 0.29) perturbations. Unlike the P1, there was no significant group differences in P2 latency bar earlier rotational P2 latencies in the CI-V than control group during rightward perturbations, *F*(1, 17.61) = 5.88, *p* = 0.026, *η*_p_^2^ = 0.25.

Given the CI-V group delays in P1 but not P2 peak latencies, the inter-peak latency was measured to assess the speed at which the CI-V group compensated for the initially delayed response (P1) to perturbations. Inter-peak latencies, P1 to P2, are plotted in Fig. 5g and h for translational and rotational motion, respectively. Translational inter-peak latency decreased with increased perturbation size for forwards (*F*(2, 1248.25) = 12.31, *p* < 0.0001, *η*_p_^2^ = 0.02) and backwards (*F*(2, 1263.27) = 48.06, *p* < 0.0001, *η*_p_^2^ = 0.07) perturbations. Participants in the CI-V group demonstrated shorter translational inter-peak latencies than control participants for medium (mean group difference = 0.17 s, *p* = 0.0002, *Cohen’s d* = 1.05, 95% CI [0.11 s, 0.43 s]) and large (mean group difference = 0.11 s, *p* = 0.0045, *Cohen’s d* = 0.84, 95% CI [0.05 s, 0.37 s]) backwards, and large left (mean group difference = 0.1 s, *p* = 0.0011, *Cohen’s d* = 0.89, 95% CI [0.08 s, 0.4 s]) perturbations. Rotational inter-peak latency increased with perturbation size for forwards (*F*(2, 736.36) = 12.39, *p* < 0.0001, *η*_p_^2^ = 0.03) perturbations, and decreased with size for backwards (*F*(2, 742.25) = 21.21, *p* < 0.0001, *η*_p_^2^ = 0.05) and left (*F*(2, 747.36) = 3.18, *p* = 0.042, *η*_p_^2^ = 0.008) perturbations. Participants in the CI-V group had larger rotational inter-peak latencies than control participants during large forwards perturbations (mean group difference = 0.23 s, *p* = 0.022, *Cohen’s d* = 0.79, 95% CI [0.07 s, 0.63 s]). Age had no effect on translational or rotational P1 latency, P2 latency, or interpeak latency for any direction (*p*’s ≥ 0.05).

### Few effects of BalanCI on response latencies

**Figure 6:**
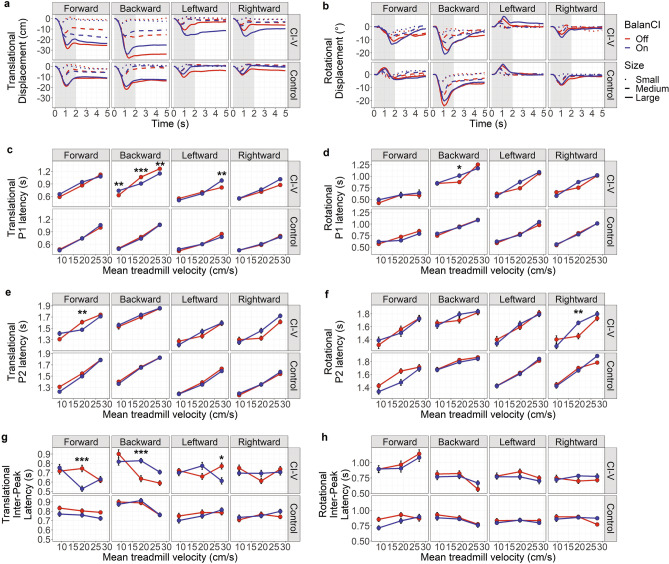
(**a**) Translational displacement (cm) over time (s) and (**b**) rotational displacement (°) over time (s) collapsed over all motion capture clusters for small, medium and large treadmill perturbations in the forwards, backwards, left and right directions for the CI-V group (n = 8) and control group (n = 15) with the BalanCI off and on. Anteroposterior axis and pitch plane are indicated for forwards and backwards perturbations, and mediolateral axis and roll plane are indicated for leftwards and rightwards perturbations. Translational motion: positive values are in the same direction as treadmill motion. Rotational motion: positive is pitch upwards and roll rightwards. The shaded region represents the region of interest for peak selection. Peak 1 latency (s) for (**c**) translational and (**d**) rotational motion across small, medium, and large treadmill perturbations, plotted as mean treadmill velocity (cm/s). Peak 2 latency (s) for (**e**) translational and (**f**) rotational motion across perturbation degrees. Inter-peak latency (P2 latency – P1 latency) for (**g**) translational and (**h**) rotational motion across perturbation degrees. All measures are collapsed over motion capture clusters. BalanCI off is in red, and on in blue. Error bars represent standard error of the mean, and points represent means. Significance differences are indicated: *p < 0.05, **p < 0.01, ***p < 0.001.

Figure 6 plots the grand-average translational motion (a) and rotational motion (b) shown in Fig. 5 with the shaded region of interest highlighted but separated for conditions in which BalanCI was off vs on. As seen in Fig. 6c, the CI-V group demonstrated later translational P1 peaks with the BalanCI on during small backwards (mean BalanCI difference = 0.11 s, *p* = 0.01, *Cohen’s d* = − 0.59, 95% CI [0.013 s, 0.2 s]) and large leftwards (mean BalanCI difference = 0.17 s, *p* = 0.0017, *Cohen’s d* = − 0.68, 95% CI [0.04 s, 0.3 s]) perturbations, but earlier P1 peaks with the BalanCI on during medium, (mean BalanCI difference = 0.16 s, *p* < 0.0001, *Cohen’s d* = 0.9, 95% CI [0.07 s, 0.25 s]), and large, (mean BalanCI difference = 0.11 s, *p* = 0.0025, *Cohen’s d* = 0.63, 95% CI [0.02 s, 0.2 s]), backwards perturbations. The BalanCI had little effect on P1 latency for rotational motion, with the CI-V group demonstrating later rotational P1 with the BalanCI on during medium backwards perturbations (mean BalanCI difference = 0.14 s, *p* = 0.04, *Cohen’s d* = − 0.68, 95% CI [0.004 s, 0.28 s]). The BalanCI had no effect on P1 latency for control participants (*p*’s > 0.05).

There was little effect of BalanCI for translational (Fig. 6e) and rotational (Fig. 6f) P2 latency. Translational P2 latency decreased with BalanCI use during forwards perturbations, *F*(1, 1254) = 6.31, *p* = 0.012, *η*_p_^2^ = 0.005, and increased with BalanCI use during rightward perturbations *F*(1, 1288.85) = 7.53, *p* = 0.006, *η*_p_^2^ = 0.006. The CI-V group had earlier translational P2 latency with the BalanCI on during medium forwards perturbations (mean BalanCI difference = 0.14 s, *p* = 0.005, *Cohen’s d* = 0.62, 95% CI [0.023 s, 0.25 s]). The CI-V group had later rotational P2 latencies with BalanCI use during medium right perturbations (mean BalanCI difference = 0.21 s, *p* = 0.0075, *Cohen’s d* = -0.79, 95% CI [0.03 s, 0.38 s]). The BalanCI had no effect on P2 latency for control participants (*p*’s > 0.05).

There was also little effect of BalanCI use for translational (Fig. 6g) and rotational (Fig. 6h) inter-peak latency. Translational inter-peak latency decreased with BalanCI use during forwards perturbations, *F*(1, 1251.78) = 15.98, *p* < 0.0001, *η*_p_^2^ = 0.01, but increased with BalanCI use during backwards perturbations, *F*(1, 1271.90) = 8.69, *p* = 0.0033, *η*_p_^2^ = 0.007. The CI-V group exhibited smaller translational inter-peak latencies with the BalanCI on during medium forwards, (mean BalanCI difference = 0.22 s, *p* < 0.0001, *Cohen’s d* = 0.91, 95% CI [0.09 s, 0.36 s]), and large left, (mean BalanCI difference = 0.16 s, *p* = 0.0092, *Cohen’s d* = 0.61, 95% CI [0.02 s, 0.3 s]), perturbations. The CI-V group exhibited larger translational inter-peak latencies with the BalanCI on during medium backwards perturbations, (mean BalanCI difference = 0.19 s, *p* = 0.0001, *Cohen’s d* = − 0.76, 95% CI [0.06 s, 0.32 s]). Rotational inter-peak latency decreased with BalanCI use during left perturbations, *F*(1, 750.12) = 4.6, *p* = 0.032, *η*_p_^2^ = 0.006. The BalanCI had no effect on inter-peak latency in control participants (*p*’s > 0.05).

### Peak amplitudes

Amplitudes were determined for the peaks described above. Amplitude findings are reflected in the AUC and are thus not explored here. ANOVA’s for peak amplitudes can be viewed in the supplementary data (Supplementary data, Tables 11–14).

## Discussion

To our knowledge, the present study is the first to examine responses to external translational perturbations in adolescents and young adults with cochleovestibular dysfunction who use CIs. Additionally, an investigative balance prosthesis, the BalanCI, was examined to determine whether it could improve stability in response to perturbations in the same cohort. We hypothesized that adolescents and young adults with cochleovestibular dysfunction who use CIs exhibit less stability than typically-developing adolescents during balance perturbations, and that BalanCI use can help improve performance on this task. Participants with bilateral cochleovestibular dysfunction demonstrated poorer postural stability and some delayed responses to perturbations compared to typically-developing peers as expected. The BalanCI supported slightly improved stability in individuals with cochleovestibular dysfunction in some perturbation conditions but could also have detrimental effects other perturbation conditions.

### Children and young adults with cochleovestibular loss perform more poorly on the BOT-2 than typically-developing children

Data shown in Fig. 2a shows that children and young adults with cochleovestibular dysfunction perform more poorly on the BOT-2 than typically developing children as measured by age- and sex- matched scores. These results are consistent with previous studies^21, 31–34^ but include a slightly older group of participants who use CIs. Given that the maximum age of the BOT-2 scaled scores is 21 years, scores from the adults who were slightly older were adjusted to these levels based on the known plateau in balance skills that occur between adolescence and age fifty^46–48^. It is also possible that scaled scores in participants over the age of 21 years are inflated as the BOT-2 age-scaled score decreases with age^30^. If so, the impaired scores in the older participants in the CI-V group were even poorer than indicated in Fig. 2a. These deficits are also evident as broken down by task, as seen in Fig. 2b and c, consistent with previous work by our group examining children in the same clinical population^26^. While the children and adults with cochleovestibular dysfunction in this study were selected due to previous findings of vestibular hypofunction, the BOT-2 confirmed significant balance deficits in these participants compared to normal.

### Children and young adults with cochleovestibular loss exhibit poorer postural stability during some degrees of perturbations than typically-developing children

Figure 3c and d plot AUC, a measure of overall stability, in children and young adults with cochleovestibular dysfunction and typically-developing children during small, medium, and large balance perturbations in four directions. Across both groups, participants demonstrated less stability with increasing perturbation sizes across all four directions. This is consistent with other studies involving perturbations of different degrees^49, 53^. The peaks selected in this study, P1 (Fig. 5c and d; first absolute peak between 0–1.5 s) and P2 (Fig. 5e and f; second absolute peak between P1 and 2 s), are consistent with the initial automatic and long-latency conscious responses to perturbations, respectively^54, 55^. Delayed peak responses to larger magnitudes of perturbations likely correspond with the increased movement required to remain upright during larger perturbations, which may take more time to peak than smaller movements.

Children with cochleovestibular dysfunction demonstrated poorer translational stability in response to medium and large backwards perturbations, larger leftward perturbations, and later latencies of translational P1 responses than typically-developing children. These measures reflect impaired “automatic” perturbation responses in children and young adults with cochleovestibular dysfunction^54, 55^. While P1 responses were delayed in the CI-V group relative to controls, P2 responses showed no significant differences between groups and this was measured as shorter P1-P2 inter-peak latencies in the CI-V group. Thus, the CI-V group had to compensate for an initially late response in order to remain upright. In general, the CI-V group may be using voluntary responses to perturbations rather than quicker automatic responses to avoid a fall in a controlled setting^56^. Without the automatic, sensory driven responses, unexpected falls may be more likely in a real-world environment.

The real-world also presents opportunities for children with balance problems. Although the CI-V group had significantly poorer BOT-2 scores, identifying balance deficits with decreased access to visual and/or proprioceptive cues, many children with cochleovestibular dysfunction participate in daily activities requiring balance including sports by compensating for loss of vestibular input with visual and proprioceptive cues^5, 57^. Together, the BOT-2 scores and the assessment of perturbations on balance in children with cochleovestibular dysfunction provide an indication of threats to balance and demonstrate how they may be compensating for their sensory deficits as they try remain upright and avoid falls.

### BalanCI use has small immediate effects on stability in children and young adults with cochleovestibular loss

Figures 4 and 6 show the effects of the BalanCI on stability and response latency, respectively, during balance perturbations in children and young adults with cochleovestibular dysfunction and typically-developing children. Some significant findings suggested a small immediate effect of the BalanCI on stability in adolescents and young adults with cochleovestibular loss. The CI-V group exhibited more translational stability with the BalanCI during large backwards perturbations (Fig. 4c) and earlier P1 peaks with the BalanCI on during medium and large backwards perturbations (Fig. 6c). These findings suggest that the BalanCI may be beneficial to individuals with SNHL who use CIs in maintaining balance through increased initial response and decreased movement during backwards perturbations. It is interesting that the CI-V group showed increased P1-P2 interpeak latencies during BalanCI use (Fig. 6g). This further reinforces a reliance on a voluntary perturbation response in the CI-V group but also shows that the BalanCI may provide some early support during the automatic phase of the perturbation response. The BalanCI also had small effects on the control group; they showed no significant latency changes with BalanCI use (Fig. 6) with subtle improvements in translational stability during large leftward perturbations and rotational stability during large rightward perturbations (Fig. 4). On the other hand, the BalanCI may have some detrimental effects on stability in other directions of perturbation. Most notably, during large sideways perturbations, the CI-V group showed less translational stability with BalanCI use, as well as delayed translational P1 responses during large leftward perturbations, and delayed rotational P1 responses for medium right perturbations. These findings contrast with those for sideways perturbations in control participants, in whom the BalanCI helped improve stability.

It is possible that differences in effects of BalanCI by direction of perturbation reflect present settings of the device. Whereas sound frequency changed with pitch of the head, head rolls create sound intensity increases in the downward leaning ear. This interaural level cue might be less available to the CI-V group than the participants in the control group given poor binaural hearing^58,59^ and potential level imbalances between the bilateral CI MAPs. Future studies and auditory balance devices should explore other auditory cues that may have more utility in individuals with poor binaural hearing, such as simple alert tones.

### Limitations

Limitations of this study include the lower number of participants in the CI-V group and their older age at testing than the control group. This reflected challenges of recruitment during the test period which occurred during early phases of the COVID-19 pandemic and strict clinical inclusion criteria for the CI-V group. To address this, age at testing was included as a fixed effect in the analyses and did not have a significant effect on reported outcomes. Moreover, the finding of significantly poorer stability in the CI-V group than controls shows that balance deficits due to cochleovestibular impairments during development remain of concern into adulthood.

In conclusion, the present study found poorer stability in children and young adults with cochleovestibular dysfunction than controls during balance perturbations. The BalanCI was able to support stability during backwards perturbations in this group but was detrimental during sideways perturbations. These findings support the potential use and feasibility of the BalanCI as a balance intervention in this group but highlights potential areas of improvement to make the auditory feedback more appropriate for populations who lack normal binaural hearing. To our knowledge, this is the first study to examine translational balance perturbations in children with cochleovestibular dysfunction. Due to the high risk of balance problems in children with cochleovestibular dysfunction, and potential consequences to implanted devices if a fall occurs, it is integral to continue studying balance tasks that mimic realistic balance threats in this population and to develop interventions to improve daily function and decrease the risk of balance related injury.

## Supplementary Information

Supplementary Tables.

## Data Availability

Data used in analyses can be made available upon request (please contact Karen A. Gordon, karen.gordon@utoronto.ca).

## References

[1] Cushing SL, Gordon KA, Rutka JA, James AL, Papsin BC (2013). Vestibular end-organ dysfunction in children with sensorineural hearing loss and cochlear implants: An expanded cohort and etiologic assessment. Otol. Neurotol..

[2] Buchman CA, Joy J, Hodges A, Telischi FF, Balkany TJ (2004). Vestibular effects of cochlear implantation. Laryngoscope.

[3] Selz PA, Girardi M, Konrad HR, Hughes LF (1996). Vestibular deficits in deaf children. Otolaryngol. Neck Surg..

[4] Inoue A (2013). Effect of vestibular dysfunction on the development of gross motor function in children with profound hearing loss. Audiol. Neurotol..

[5] Kaga K (1999). Vestibular compensation in infants and children with congenital and acquired vestibular loss in both ears. Int. J. Pediatr. Otorhinolaryngol..

[6] Rine RM (2000). Evidence of progressive delay of motor development in children with sensorineural hearing loss and concurrent vestibular dysfunction. Percept. Mot. Skills.

[7] Wolter NE, Gordon KA, Papsin BC, Cushing SL (2015). Vestibular and balance impairment contributes to cochlear implant failure in children. Otol. Neurotol..

[8] Allum JHJ, Honegger F (2017). Vibro-tactile and auditory balance biofeedback changes muscle activity patterns: Possible implications for vestibular implants. J. Vestib. Res..

[9] Ghulyan-Bedikian V, Paolino M, Paolino F (2013). Short-term retention effect of rehabilitation using head position-based electrotactile feedback to the tongue: Influence of vestibular loss and old-age. Gait Posture.

[10] Kingma H (2019). Vibrotactile feedback improves balance and mobility in patients with severe bilateral vestibular loss. J. Neurol..

[11] Peterka RJ, Wall C, Kentala E (2006). Determining the effectiveness of a vibrotactile balance prosthesis. J. Vestib. Res..

[12] Wall C, Kentala E (2005). Control of sway using vibrotactile feedback of body tilt in patients with moderate and severe postural control deficits. J. Vestib. Res..

[13] Kentala E, Vivas J, Wall C (2003). Reduction of postural sway by use of a vibrotactile balance prosthesis prototype in subjects with vestibular deficits. Ann. Otol. Rhinol. Laryngol..

[14] Sienko K, Balkwill M, Oddsson L, Wall C (2009). Effects of multi-directional vibrotactile feedback on vestibular-deficient postural performance during continuous multi-directional support surface perturbations. J. Vestib. Res..

[15] Sienko K, Whitney S, Carender W, Wall C (2017). The role of sensory augmentation for people with vestibular deficits: Real-time balance aid and/or rehabilitation device?. J. Vestib. Res..

[16] Bao T (2019). Effects of long-term vestibular rehabilitation therapy with vibrotactile sensory augmentation for people with unilateral vestibular disorders–A randomized preliminary study. J. Vestib. Res..

[17] Basta D, Singbartl F, Todt I, Clarke A, Ernst A (2008). Vestibular rehabilitation by auditory feedback in otolith disorders. Gait Posture.

[18] Chiari L (2005). Audio-biofeedback for balance improvement: An accelerometry-based system. IEEE Trans. Biomed. Eng..

[19] Dozza M, Chiari L, Horak FB (2005). Audio-biofeedback improves balance in patients with bilateral vestibular loss. Arch. Phys. Med. Rehabil..

[20] Honegger F, Hillebrandt IM, van den Elzen NG, Tang K-S, Allum JH (2013). The effect of prosthetic feedback on the strategies and synergies used by vestibular loss subjects to control stance. J. NeuroEng. Rehabil..

[21] Cushing SL, Chia R, James AL, Papsin BC, Gordon KA (2008). A test of static and dynamic balance function in children with cochlear implants: The vestibular olympics. Arch. Otolaryngol. Neck Surg..

[22] Mazaheryazdi M, Moossavi A, Sarrafzadah J, Talebian S, Jalaie S (2017). Study of the effects of hearing on static and dynamic postural function in children using cochlear implants. Int. J. Pediatr. Otorhinolaryngol..

[23] Oikawa K, Kobayashi Y, Hiraumi H, Yonemoto K, Sato H (2018). Body balance function of cochlear implant patients with and without sound conditions. Clin. Neurophysiol..

[24] Shayman CS, Mancini M, Weaver TS, King LA, Hullar TE (2018). The contribution of cochlear implants to postural stability. Laryngoscope.

[25] Campos J, Ramkhalawansingh R, Pichora-Fuller MK (2018). Hearing, self-motion perception, mobility, and aging. Hear. Res..

[26] Wolter NE (2020). BalanCI: Head-referenced cochlear implant stimulation improves balance in children with bilateral cochleovestibular loss. Audiol. Neurotol..

[27] Parkes WJ (2017). Vestibular evoked myogenic potential testing as an objective measure of vestibular stimulation with cochlear implants. Laryngoscope.

[28] Cushing SL, Papsin BC, Gordon KA (2006). Incidence and characteristics of facial nerve stimulation in children with cochlear implants. Laryngoscope.

[29] Cushing SL, Papsin BC, Strantzas S, Gordon KA (2009). Facial nerve electromyography: A useful tool in detecting nonauditory side effects of cochlear implantation. J. Otolaryngol. Neck Surg..

[30] Bruininks RH, Bruininks BD (2005). BOT2: Bruininks-Oseretsky Test of Motor Proficiency.

[31] Cushing SL, Papsin BC, Rutka JA, James AL, Gordon KA (2008). Evidence of vestibular and balance dysfunction in children with profound sensorineural hearing loss using cochlear implants. Laryngoscope.

[32] Eustaquio ME, Berryhill W, Wolfe JA, Saunders JE (2011). Balance in children with bilateral cochlear implants. Otol. Neurotol..

[33] McSweeny C, Cushing SL, Campos JL, Papsin BC, Gordon KA (2021). Functional consequences of poor binaural hearing in development: evidence from children with unilateral hearing loss and children receiving bilateral cochlear implants. Trends Hear..

[34] Wolter NE (2021). Impact of the sensory environment on balance in children with bilateral cochleovestibular loss. Hear. Res..

[35] Hatzitaki V, Amiridis IG, Arabatzi F (2005). Aging effects on postural responses to self-imposed balance perturbations. Gait Posture.

[36] Kanekar N, Aruin AS (2014). Aging and balance control in response to external perturbations: Role of anticipatory and compensatory postural mechanisms. Age.

[37] Kurz I (2016). Unexpected perturbations training improves balance control and voluntary stepping times in older adults—A double blind randomized control trial. BMC Geriatr..

[38] Carpenter MG, Allum JHJ, Honegger F, Adkin AL, Bloem BR (2004). Postural abnormalities to multidirectional stance perturbations in Parkinson’s disease. J. Neurol. Neurosurg. Psychiatry.

[39] Kuhman DJ, Walker HC, Hurt CP (2020). Dopamine-mediated improvements in dynamic balance control in Parkinson’s disease. Gait Posture.

[40] Nanhoe-Mahabier W (2012). First trial reactions and habituation rates over successive balance perturbations in Parkinson’s disease. Neuroscience.

[41] Esmaeili V (2020). Intense and unpredictable perturbations during gait training improve dynamic balance abilities in chronic hemiparetic individuals: A randomized controlled pilot trial. J. NeuroEng. Rehabil..

[42] Handelzalts S (2019). Effects of perturbation-based balance training in subacute persons with stroke: A randomized controlled trial. Neurorehabil. Neural Repair.

[43] Bernard-Demanze L (2014). Static and dynamic posture control in postlingual cochlear implanted patients: Effects of dual-tasking, visual and auditory inputs suppression. Front. Integr. Neurosci..

[44] Shupert CL, Horak FB (1996). Effects of vestibular loss on head stabilization in response to head and body perturbations. J. Vestib. Res..

[45] Winkler PA, Esses B (2011). Platform tilt perturbation as an intervention for people with chronic vestibular dysfunction. J. Neurol. Phys. Ther..

[46] Goble DJ, Baweja HS (2018). Normative data for the BTrackS balance test of postural sway: Results from 16,357 community-dwelling individuals who were 5 to 100 years old. Phys. Ther..

[47] Riis J (2020). Lifespan data on postural balance in multiple standing positions. Gait Posture.

[48] Sheldon J (1963). The effect of age on the control of sway. Gerontol. Clin. (Basel).

[49] Brauer SG, Woollacott M, Shumway-Cook A (2002). The influence of a concurrent cognitive task on the compensatory stepping response to a perturbation in balance-impaired and healthy elders. Gait Posture.

[50] Diener HC, Horak FB, Nashner LM (1988). Influence of stimulus parameters on human postural responses. J. Neurophysiol..

[51] Rogers MW, Hain TC, Hanke TA, Janssen I (1996). Stimulus parameters and inertial load: Effects on the incidence of protective stepping responses in healthy human subjects. Arch. Phys. Med. Rehabil..

[52] Schielzeth H (2020). Robustness of linear mixed-effects models to violations of distributional assumptions. Methods Ecol. Evol..

[53] McIlroy WE, Maki BE (1993). Task constraints on foot movement and the incidence of compensatory stepping following perturbation of upright stance. Brain Res..

[54] Norrie RG, Maki BE, Staines WR, McIlroy WE (2002). The time course of attention shifts following perturbation of upright stance. Exp. Brain Res..

[55] Rogers MW, Mille M-L (2018). Balance perturbations. Handb. Clin. Neurol..

[56] Horak FB, Henry SM, Shumway-Cook A (1997). Postural perturbations: New insights for treatment of balance disorders. Phys. Ther..

[57] Kaga K, Shinjo Y, Jin Y, Takegoshi H (2008). Vestibular failure in children with congenital deafness. Int. J. Audiol..

[58] Gordon KA, Deighton MR, Abbasalipour P, Papsin BC (2014). Perception of binaural cues develops in children who are deaf through bilateral cochlear implantation. PLoS One.

[59] Fitzgerald MB, Kan A, Goupell MJ (2015). Bilateral loudness balancing and distorted spatial perception in recipients of bilateral cochlear implants. Ear Hear..

