# Supplementary material for: Evaluating the use of a balance prosthesis during balance perturbations in children and young adults with cochleovestibular dysfunction
Source: Sci Rep. 2023 Jun 15;13:9721. doi: 10.1038/s41598-023-36613-3 (PMC10272120; doi:10.1038/s41598-023-36613-3)
Supplement: Supplementary file 1 — Supplementary Tables. [file 41598_2023_36613_MOESM1_ESM.pdf]

Evaluating the use of a balance prosthesis during balance perturbations in children and young adults with cochleovestibular dysfunction

Rebecca S. Benjamin[1,2], Sharon L. Cushing[1,2,3,4], Alan W. Blakeman[2], Jennifer L. Campos[5,6], Blake C. Papsin[1,2,3,4], Karen A. Gordon*[1,2,3,4,7]

[1]Institute of Medical Sciences, University of Toronto, Toronto, ON, Canada
[2]Archie's Cochlear Implant Laboratory, Hospital for Sick Children, Toronto, ON, Canada
[3]Department of Otolaryngology, Head and Neck Surgery, Hospital for Sick Children, Toronto, ON, Canada
[4]Department of Otolaryngology, Head and Neck Surgery, University of Toronto, Toronto, ON, Canada
[5]Department of Psychology, University of Toronto, Toronto, ON, Canada
[6]KITE, Toronto Rehabilitation Institute, University Health Network, Toronto, ON, Canada
[7]Department of Communication Disorders, Hospital for Sick Children, Toronto, ON, Canada

*Corresponding author: Karen A. Gordon, karen-a.gordon@sickkids.ca

Supplementary Data

Table 1.
Static BOT-2 tasks ANOVA.

	Sum Sq	Mean Sq	NumDF	DenDF	F value	Pr(>F)
**Group**	337.48	337.48	1.00	19.00	104.56	3.67e-09
**`Task Number`**	406.24	67.71	6.00	126.00	20.98	6.01e-17
**Sex**	3.60	3.60	1.00	19.00	1.12	0.30
**Age**	8.91	8.91	1.00	19.00	2.76	0.11
**Group:`Task Number`**	128.28	21.38	6.00	126.00	6.62	4.16e-06

Note: Model: value ~ Group*`Task Number` + Sex + Age + (1|`Study ID`). ANOVA using Satterthwaite's method. Degrees-of-freedom adjusted for multiple comparisons using Kenward-Roger method.

Table 2.
Dynamic BOT-2 tasks ANOVA.

	Sum Sq	Mean Sq	NumDF	DenDF	F value	Pr(>F)
**Group**	2.85	2.85	1.00	19.00	8.87	0.01
**`Task Number`**	1.47	1.47	1.00	21.00	4.57	0.04
**Sex**	0.01	0.01	1.00	19.00	0.02	0.89
**Age**	0.99	0.99	1.00	19.00	3.07	0.10

**Group:`Task Number`**	1.47	1.47	1.00	21.00	4.57	0.04

Note: Model: value ~ Group*`Task Number` + Sex + Age + (1|`Study ID`). ANOVA using Satterthwaite's method. Degrees-of-freedom adjusted for multiple comparisons using Kenward-Roger method.

Table 3.
Translational area under the curve ANOVA.

A) Forwards perturbations:

	**Sum Sq**	**Mean Sq**	**NumDF**	**DenDF**	**F value**	**Pr(>F)**
**BCI**	5095.51	5095.51	1.00	1250.80	3.76	0.05
**Size**	906944.64	453472.32	2.00	1248.25	334.52	4.68e-117
**Group**	4154.37	4154.37	1.00	21.48	3.06	0.09
**Marker**	38544.96	9636.24	4.00	1247.97	7.11	1.17e-05
**Age**	4580.33	4580.33	1.00	17.80	3.38	0.08
**Trial_order**	374.50	374.50	1.00	1110.51	0.28	0.60
**Sex**	2707.16	2707.16	1.00	17.88	2.00	0.17
**Height**	5174.72	5174.72	1.00	17.85	3.82	0.07
**BCI:Size**	18574.07	9287.03	2.00	1248.44	6.85	0.00
**BCI:Group**	723.78	723.78	1.00	1250.48	0.53	0.47
**Size:Group**	48191.46	24095.73	2.00	1248.37	17.78	2.44e-08
**BCI:Marker**	11074.61	2768.65	4.00	1247.95	2.04	0.09
**Size:Marker**	10253.86	1281.73	8.00	1247.95	0.95	0.48
**Group:Marker**	23399.15	5849.79	4.00	1247.97	4.32	1.80e-03
**BCI:Size:Group**	16498.11	8249.06	2.00	1248.75	6.09	2.34e-03
**BCI:Size:Marker**	2196.55	274.57	8.00	1247.95	0.20	0.99
**BCI:Group:Marker**	7792.51	1948.13	4.00	1247.95	1.44	0.22
**Size:Group:Marker**	6777.78	847.22	8.00	1247.95	0.62	0.76
**BCI:Size:Group:Marker**	3462.94	432.87	8.00	1247.95	0.32	0.96

B) Backwards perturbations:

	**Sum Sq**	**Mean Sq**	**NumDF**	**DenDF**	**F value**	**Pr(>F)**
**BCI**	18057.66	18057.66	1.00	1269.17	14.05	1.86e-04
**Size**	1530151.06	765075.53	2.00	1263.72	595.42	6.63e-183
**Group**	35880.77	35880.77	1.00	22.13	27.92	2.60e-05

**Marker**	172924.63	43231.16	4.00	1263.73	33.64	1.05e-26
**Age**	2479.78	2479.78	1.00	17.18	1.93	0.18
**Trial_order**	333.29	333.29	1.00	910.52	0.26	0.61
**Sex**	443.18	443.18	1.00	17.09	0.34	0.56
**Height**	404.63	404.63	1.00	17.29	0.31	0.58
**BCI:Size**	27483.32	13741.66	2.00	1264.27	10.69	2.48e-05
**BCI:Group**	11011.99	11011.99	1.00	1268.46	8.57	3.48e-03
**Size:Group**	148486.36	74243.18	2.00	1264.60	57.78	9.72e-25
**BCI:Marker**	2886.96	721.74	4.00	1263.43	0.56	0.69
**Size:Marker**	47947.40	5993.43	8.00	1263.40	4.66	1.22e-05
**Group:Marker**	12755.69	3188.92	4.00	1263.73	2.48	0.04
**BCI:Size:Group**	10947.42	5473.71	2.00	1264.92	4.26	0.01
**BCI:Size:Marker**	6959.28	869.91	8.00	1263.40	0.68	0.71
**BCI:Group:Marker**	3603.73	900.93	4.00	1263.43	0.70	0.59
**Size:Group:Marker**	21526.48	2690.81	8.00	1263.40	2.09	0.03
**BCI:Size:Group:Marker**	7064.19	883.02	8.00	1263.40	0.69	0.70

C) Left perturbations:

	**Sum Sq**	**Mean Sq**	**NumDF**	**DenDF**	**F value**	**Pr(>F)**
**BCI**	1460.13	1460.13	1.00	1274.39	2.08	0.15
**Size**	177167.92	88583.96	2.00	1272.48	126.03	1.16e-50
**Group**	5165.92	5165.92	1.00	19.43	7.35	0.01
**Marker**	48280.97	12070.24	4.00	1271.82	17.17	9.98e-14
**Age**	56.14	56.14	1.00	17.71	0.08	0.78
**Trial_order**	340.87	340.87	1.00	1254.15	0.48	0.49
**Sex**	1857.91	1857.91	1.00	17.72	2.64	0.12
**Height**	190.32	190.32	1.00	17.73	0.27	0.61
**BCI:Size**	11460.76	5730.38	2.00	1272.75	8.15	3.03e-04
**BCI:Group**	17343.17	17343.17	1.00	1273.75	24.67	7.71e-07
**Size:Group**	22301.02	11150.51	2.00	1272.14	15.86	1.57e-07
**BCI:Marker**	116.16	29.04	4.00	1271.80	0.04	1.00
**Size:Marker**	11261.68	1407.71	8.00	1271.79	2.00	0.04
**Group:Marker**	4376.84	1094.21	4.00	1271.82	1.56	0.18
**BCI:Size:Group**	42463.54	21231.77	2.00	1272.05	30.21	1.53e-13
**BCI:Size:Marker**	974.81	121.85	8.00	1271.80	0.17	0.99
**BCI:Group:Marker**	294.32	73.58	4.00	1271.80	0.10	0.98
**Size:Group:Marker**	1261.27	157.66	8.00	1271.79	0.22	0.99
**BCI:Size:Group:Marker**	845.07	105.63	8.00	1271.80	0.15	1.00

D) Right perturbations:

	**Sum Sq**	**Mean Sq**	**NumDF**	**DenDF**	**F value**	**Pr(>F)**
**BCI**	841.34	841.34	1.00	1284.25	1.50	0.22

**Size**	164668.10	82334.05	2.00	1283.41	147.08	3.09e-58
**Group**	1364.56	1364.56	1.00	19.17	2.44	0.13
**Marker**	62508.03	15627.01	4.00	1282.93	27.92	2.95e-22
**Age**	78.67	78.67	1.00	17.85	0.14	0.71
**Trial_order**	4454.25	4454.25	1.00	1297.51	7.96	4.86e-03
**Sex**	581.10	581.10	1.00	17.87	1.04	0.32
**Height**	34.03	34.03	1.00	17.88	0.06	0.81
**BCI:Size**	1555.32	777.66	2.00	1283.05	1.39	0.25
**BCI:Group**	6641.14	6641.14	1.00	1284.04	11.86	5.91e-04
**Size:Group**	29330.19	14665.10	2.00	1283.20	26.20	7.06e-12
**BCI:Marker**	731.19	182.80	4.00	1282.93	0.33	0.86
**Size:Marker**	18094.02	2261.75	8.00	1282.93	4.04	9.33e-05
**Group:Marker**	1862.17	465.54	4.00	1282.93	0.83	0.51
**BCI:Size:Group**	12869.41	6434.70	2.00	1283.12	11.49	1.13e-05
**BCI:Size:Marker**	5127.30	640.91	8.00	1282.93	1.14	0.33
**BCI:Group:Marker**	838.17	209.54	4.00	1282.93	0.37	0.83
**Size:Group:Marker**	5789.03	723.63	8.00	1282.93	1.29	0.24
**BCI:Size:Group:Marker**	2333.95	291.74	8.00	1282.93	0.52	0.84

Note: Model: AUC~BCI*Size*Group*Marker + Age + Trial_order + Sex + Height + (1|`Study.code`). Degrees-of-freedom adjusted for multiple comparisons using Kenward-Roger method.

Table 4.
Rotational area under the curve ANOVA.
A) Forwards perturbations:

	**Sum Sq**	**Mean Sq**	**NumDF**	**DenDF**	**F value**	**Pr(>F)**
**BCI**	452.93	452.93	1.00	713.09	0.80	0.37
**Size**	32625.73	16312.87	2.00	711.05	28.80	9.44e-13
**Group**	1879.15	1879.15	1.00	21.76	3.32	0.08
**Marker**	69329.50	34664.75	2.00	710.95	61.19	3.02e-25
**Age**	401.41	401.41	1.00	17.49	0.71	0.41
**Trial_order**	1206.57	1206.57	1.00	671.86	2.13	0.14
**Sex**	200.92	200.92	1.00	17.63	0.35	0.56
**Height**	669.48	669.48	1.00	17.59	1.18	0.29
**BCI:Size**	1420.91	710.46	2.00	711.25	1.25	0.29
**BCI:Group**	168.82	168.82	1.00	712.68	0.30	0.59
**Size:Group**	5302.86	2651.43	2.00	711.07	4.68	0.01
**BCI:Marker**	291.78	145.89	2.00	710.86	0.26	0.77
**Size:Marker**	8833.91	2208.48	4.00	710.81	3.90	3.86e-03
**Group:Marker**	4844.94	2422.47	2.00	710.92	4.28	0.01
**BCI:Size:Group**	2342.59	1171.29	2.00	711.32	2.07	0.13
**BCI:Size:Marker**	399.56	99.89	4.00	710.77	0.18	0.95
**BCI:Group:Marker**	18.00	9.00	2.00	710.82	0.02	0.98
**Size:Group:Marker**	5984.70	1496.18	4.00	710.79	2.64	0.03
**BCI:Size:Group:Marker**	7736.07	1934.02	4.00	710.77	3.41	0.01

B) Backwards perturbations:

	Sum Sq	Mean Sq	NumDF	DenDF	F value	Pr(>F)
**BCI**	202.55	202.55	1.00	742.35	0.27	0.60
**Size**	142792.88	71396.44	2.00	739.76	95.54	1.24e-37
**Group**	365.60	365.60	1.00	20.98	0.49	0.49
**Marker**	48786.63	24393.31	2.00	740.08	32.64	2.60e-14
**Age**	1056.32	1056.32	1.00	17.40	1.41	0.25
**Trial_order**	139.42	139.42	1.00	713.77	0.19	0.67
**Sex**	281.36	281.36	1.00	17.33	0.38	0.55
**Height**	1184.14	1184.14	1.00	17.53	1.58	0.22
**BCI:Size**	774.18	387.09	2.00	739.97	0.52	0.60
**BCI:Group**	3543.41	3543.41	1.00	741.97	4.74	0.03
**Size:Group**	4099.26	2049.63	2.00	740.12	2.74	0.07
**BCI:Marker**	622.70	311.35	2.00	739.66	0.42	0.66
**Size:Marker**	6854.21	1713.55	4.00	739.62	2.29	0.06
**Group:Marker**	2547.84	1273.92	2.00	740.08	1.70	0.18
**BCI:Size:Group**	781.17	390.58	2.00	740.25	0.52	0.59
**BCI:Size:Marker**	321.05	80.26	4.00	739.62	0.11	0.98
**BCI:Group:Marker**	1051.58	525.79	2.00	739.66	0.70	0.50
**Size:Group:Marker**	265.99	66.50	4.00	739.62	0.09	0.99
**BCI:Size:Group:Marker**	801.59	200.40	4.00	739.62	0.27	0.90

C) Left perturbations:

	Sum Sq	Mean Sq	NumDF	DenDF	F value	Pr(>F)
**BCI**	165.23	165.23	1.00	720.43	1.19	0.28
**Size**	14650.09	7325.04	2.00	717.22	52.72	4.34e-22
**Group**	100.49	100.49	1.00	21.09	0.72	0.40
**Marker**	6487.13	3243.57	2.00	716.04	23.35	1.51e-10
**Age**	38.56	38.56	1.00	16.33	0.28	0.61
**Trial_order**	40.63	40.63	1.00	582.78	0.29	0.59
**Sex**	124.91	124.91	1.00	16.47	0.90	0.36
**Height**	228.35	228.35	1.00	16.46	1.64	0.22
**BCI:Size**	1768.14	884.07	2.00	718.28	6.36	1.82e-03
**BCI:Group**	1057.25	1057.25	1.00	719.92	7.61	0.01
**Size:Group**	48.15	24.08	2.00	716.59	0.17	0.84
**BCI:Marker**	14.53	7.26	2.00	715.75	0.05	0.95
**Size:Marker**	1108.21	277.05	4.00	715.77	1.99	0.09
**Group:Marker**	25.28	12.64	2.00	716.03	0.09	0.91
**BCI:Size:Group**	650.21	325.10	2.00	717.15	2.34	0.10
**BCI:Size:Marker**	759.65	189.91	4.00	715.78	1.37	0.24
**BCI:Group:Marker**	331.31	165.65	2.00	715.76	1.19	0.30
**Size:Group:Marker**	50.83	12.71	4.00	715.78	0.09	0.99
**BCI:Size:Group:Marker**	280.51	70.13	4.00	715.76	0.50	0.73

D) Right perturbations:

	**Sum Sq**	**Mean Sq**	**NumDF**	**DenDF**	**F value**	**Pr(>F)**
**BCI**	182.03	182.03	1.00	759.96	0.89	0.35
**Size**	12290.45	6145.22	2.00	757.60	30.02	2.84e-13
**Group**	774.30	774.30	1.00	24.02	3.78	0.06
**Marker**	8432.49	4216.25	2.00	756.22	20.60	1.95e-09
**Age**	626.32	626.32	1.00	17.90	3.06	0.10
**Trial_order**	193.28	193.28	1.00	629.44	0.94	0.33
**Sex**	151.01	151.01	1.00	17.88	0.74	0.40
**Height**	711.49	711.49	1.00	17.96	3.48	0.08
**BCI:Size**	384.99	192.50	2.00	756.71	0.94	0.39
**BCI:Group**	1633.52	1633.52	1.00	758.82	7.98	4.86e-03
**Size:Group**	1796.31	898.16	2.00	757.12	4.39	0.01
**BCI:Marker**	271.28	135.64	2.00	756.19	0.66	0.52
**Size:Marker**	830.16	207.54	4.00	756.19	1.01	0.40
**Group:Marker**	261.36	130.68	2.00	756.21	0.64	0.53
**BCI:Size:Group**	3630.78	1815.39	2.00	756.76	8.87	1.56e-04
**BCI:Size:Marker**	852.03	213.01	4.00	756.20	1.04	0.39
**BCI:Group:Marker**	22.96	11.48	2.00	756.19	0.06	0.95
**Size:Group:Marker**	873.27	218.32	4.00	756.19	1.07	0.37
**BCI:Size:Group:Marker**	1310.54	327.63	4.00	756.21	1.60	0.17

Note: Model: AUC~BCI*Size*Group*Marker + Age + Trial_order + Sex + Height + (1|`Study.code`). ANOVA using Satterthwaite's method. Degrees-of-freedom adjusted for multiple comparisons using Kenward-Roger method.

Table 5.
Translational P1 latency ANOVA.
A) Forwards perturbations:

	**Sum Sq**	**Mean Sq**	**NumDF**	**DenDF**	**F value**	**Pr(>F)**
**BCI**	0.16	0.16	1.00	1250.87	5.06	0.02
**Size**	56.34	28.17	2.00	1247.77	873.10	7.14e-238
**Group**	0.05	0.05	1.00	21.48	1.53	0.23
**Marker**	3.45	0.86	4.00	1247.42	26.75	2.55e-21
**Age**	0.06	0.06	1.00	17.23	1.74	0.20
**Trial_order**	0.30	0.30	1.00	1028.63	9.15	2.55e-03
**Sex**	0.13	0.13	1.00	17.33	4.15	0.06
**Height**	0.02	0.02	1.00	17.29	0.71	0.41
**BCI:Size**	0.03	0.01	2.00	1248.00	0.46	0.63
**BCI:Group**	0.05	0.05	1.00	1250.48	1.63	0.20
**Size:Group**	0.50	0.25	2.00	1247.91	7.72	4.66e-04
**BCI:Marker**	0.36	0.09	4.00	1247.39	2.77	0.03
**Size:Marker**	0.32	0.04	8.00	1247.39	1.24	0.27
**Group:Marker**	0.48	0.12	4.00	1247.42	3.70	0.01
**BCI:Size:Group**	0.52	0.26	2.00	1248.37	8.01	3.50e-04

**BCI:Size:Marker**	0.21	0.03	8.00	1247.39	0.82	0.58
**BCI:Group:Marker**	0.23	0.06	4.00	1247.39	1.80	0.13
**Size:Group:Marker**	0.14	0.02	8.00	1247.39	0.56	0.81
**BCI:Size:Group:Marker**	0.08	0.01	8.00	1247.39	0.29	0.97

B) Backwards perturbations:

	**Sum Sq**	**Mean Sq**	**NumDF**	**DenDF**	**F value**	**Pr(>F)**
**BCI**	0.38	0.38	1.00	1268.81	12.23	4.87e-04
**Size**	61.35	30.67	2.00	1263.76	987.85	4.81e-259
**Group**	0.81	0.81	1.00	21.85	26.23	4.01e-05
**Marker**	7.58	1.90	4.00	1263.77	61.04	3.48e-47
**Age**	0.01	0.01	1.00	17.26	0.17	0.68
**Trial_order**	0.20	0.20	1.00	964.92	6.30	0.01
**Sex**	0.04	0.04	1.00	17.18	1.31	0.27
**Height**	0.05	0.05	1.00	17.36	1.46	0.24
**BCI:Size**	1.14	0.57	2.00	1264.26	18.40	1.33e-08
**BCI:Group**	0.13	0.13	1.00	1268.13	4.06	0.04
**Size:Group**	0.40	0.20	2.00	1264.57	6.45	1.64e-03
**BCI:Marker**	0.04	0.01	4.00	1263.50	0.28	0.89
**Size:Marker**	0.65	0.08	8.00	1263.47	2.62	0.01
**Group:Marker**	1.05	0.26	4.00	1263.77	8.45	1.00e-06
**BCI:Size:Group**	0.91	0.45	2.00	1264.86	14.64	5.19e-07
**BCI:Size:Marker**	0.23	0.03	8.00	1263.47	0.94	0.48
**BCI:Group:Marker**	0.16	0.04	4.00	1263.50	1.26	0.29
**Size:Group:Marker**	0.24	0.03	8.00	1263.47	0.96	0.46
**BCI:Size:Group:Marker**	0.13	0.02	8.00	1263.47	0.54	0.83

C) Left perturbations:

	**Sum Sq**	**Mean Sq**	**NumDF**	**DenDF**	**F value**	**Pr(>F)**
**BCI**	0.08	0.08	1.00	1277.08	1.28	0.26
**Size**	26.87	13.44	2.00	1273.95	216.11	1.54e-81
**Group**	0.28	0.28	1.00	20.56	4.54	0.05
**Marker**	4.10	1.02	4.00	1272.85	16.48	3.55e-13
**Age**	0.01	0.01	1.00	17.66	0.21	0.65
**Trial_order**	0.20	0.20	1.00	1130.67	3.29	0.07
**Sex**	0.02	0.02	1.00	17.67	0.30	0.59
**Height**	0.01	0.01	1.00	17.70	0.19	0.67
**BCI:Size**	0.24	0.12	2.00	1274.39	1.96	0.14
**BCI:Group**	0.04	0.04	1.00	1276.06	0.67	0.41
**Size:Group**	0.02	0.01	2.00	1273.38	0.12	0.88
**BCI:Marker**	0.15	0.04	4.00	1272.82	0.61	0.65
**Size:Marker**	6.40	0.80	8.00	1272.80	12.87	6.18e-18
**Group:Marker**	0.44	0.11	4.00	1272.85	1.78	0.13
**BCI:Size:Group**	1.60	0.80	2.00	1273.25	12.90	2.85e-06
**BCI:Size:Marker**	0.82	0.10	8.00	1272.80	1.64	0.11

**BCI:Group:Marker**	0.08	0.02	4.00	1272.82	0.34	0.85
**Size:Group:Marker**	0.09	0.01	8.00	1272.80	0.18	0.99
**BCI:Size:Group:Marker**	0.68	0.09	8.00	1272.80	1.37	0.21

D) Right perturbations:

	**Sum Sq**	**Mean Sq**	**NumDF**	**DenDF**	**F value**	**Pr(>F)**
**BCI**	0.43	0.43	1.00	1287.46	6.89	0.01
**Size**	27.93	13.96	2.00	1285.80	224.38	2.58e-84
**Group**	0.07	0.07	1.00	20.32	1.06	0.31
**Marker**	4.63	1.16	4.00	1284.81	18.62	6.90e-15
**Age**	0.01	0.01	1.00	17.64	0.09	0.77
**Trial_order**	0.20	0.20	1.00	1235.51	3.24	0.07
**Sex**	0.01	0.01	1.00	17.69	0.13	0.72
**Height**	2.32e-03	2.32e-03	1.00	17.72	0.04	0.85
**BCI:Size**	0.15	0.08	2.00	1285.05	1.23	0.29
**BCI:Group**	0.20	0.20	1.00	1287.02	3.15	0.08
**Size:Group**	0.16	0.08	2.00	1285.38	1.31	0.27
**BCI:Marker**	0.05	0.01	4.00	1284.81	0.20	0.94
**Size:Marker**	5.79	0.72	8.00	1284.81	11.63	4.61e-16
**Group:Marker**	0.06	0.01	4.00	1284.81	0.24	0.92
**BCI:Size:Group**	0.31	0.16	2.00	1285.20	2.51	0.08
**BCI:Size:Marker**	0.21	0.03	8.00	1284.81	0.43	0.90
**BCI:Group:Marker**	0.30	0.07	4.00	1284.81	1.19	0.31
**Size:Group:Marker**	0.31	0.04	8.00	1284.81	0.63	0.76
**BCI:Size:Group:Marker**	0.54	0.07	8.00	1284.81	1.08	0.37

Note: Model: Pert.Time_X~BCI*Size*Group*Marker + Age + Trial_order + Sex + Height + (1|`Study.code`). ANOVA using Satterthwaite's method. Degrees-of-freedom adjusted for multiple comparisons using Kenward-Roger method.

Table 6.
Rotational P1 latency ANOVA.

A) Forwards perturbations:

	**Sum Sq**	**Mean Sq**	**NumDF**	**DenDF**	**F value**	**Pr(>F)**
**BCI**	2.01e-06	2.01e-06	1.00	738.22	2.42e-05	1.00
**Size**	4.44	2.22	2.00	736.42	26.80	5.83e-12
**Group**	0.21	0.21	1.00	17.98	2.55	0.13
**Marker**	5.53	2.76	2.00	736.22	33.40	1.30e-14
**Age**	0.01	0.01	1.00	18.03	0.16	0.70
**Trial_order**	0.56	0.56	1.00	737.89	6.76	0.01
**Sex**	0.06	0.06	1.00	18.03	0.71	0.41
**Height**	0.07	0.07	1.00	18.15	0.84	0.37
**BCI:Size**	0.36	0.18	2.00	736.36	2.18	0.11
**BCI:Group**	0.51	0.51	1.00	737.89	6.21	0.01
**Size:Group**	0.35	0.18	2.00	736.40	2.12	0.12

**BCI:Marker**	0.20	0.10	2.00	736.15	1.21	0.30
**Size:Marker**	1.07	0.27	4.00	736.16	3.23	0.01
**Group:Marker**	1.29	0.65	2.00	736.22	7.79	4.47e-04
**BCI:Size:Group**	0.06	0.03	2.00	736.34	0.37	0.69
**BCI:Size:Marker**	0.08	0.02	4.00	736.15	0.24	0.92
**BCI:Group:Marker**	0.48	0.24	2.00	736.15	2.90	0.06
**Size:Group:Marker**	0.30	0.07	4.00	736.16	0.89	0.47
**BCI:Size:Group:Marker**	0.28	0.07	4.00	736.15	0.86	0.49

B) Backwards perturbations:

	**Sum Sq**	**Mean Sq**	**NumDF**	**DenDF**	**F value**	**Pr(>F)**
**BCI**	0.03	0.03	1.00	745.38	0.60	0.44
**Size**	13.94	6.97	2.00	741.58	156.73	1.69e-57
**Group**	0.03	0.03	1.00	16.96	0.76	0.39
**Marker**	5.11	2.56	2.00	742.54	57.51	6.02e-24
**Age**	0.04	0.04	1.00	17.18	0.95	0.34
**Trial_order**	1.23	1.23	1.00	745.93	27.57	1.98e-07
**Sex**	0.08	0.08	1.00	17.00	1.75	0.20
**Height**	2.51e-04	2.51e-04	1.00	17.52	0.01	0.94
**BCI:Size**	0.32	0.16	2.00	741.84	3.64	0.03
**BCI:Group**	0.09	0.09	1.00	744.85	1.92	0.17
**Size:Group**	0.43	0.22	2.00	741.58	4.84	0.01
**BCI:Marker**	0.12	0.06	2.00	741.52	1.31	0.27
**Size:Marker**	0.12	0.03	4.00	741.43	0.70	0.59
**Group:Marker**	0.40	0.20	2.00	742.54	4.48	0.01
**BCI:Size:Group**	0.45	0.22	2.00	741.83	5.03	0.01
**BCI:Size:Marker**	0.18	0.05	4.00	741.43	1.03	0.39
**BCI:Group:Marker**	0.04	0.02	2.00	741.52	0.47	0.62
**Size:Group:Marker**	0.09	0.02	4.00	741.43	0.53	0.72
**BCI:Size:Group:Marker**	0.27	0.07	4.00	741.43	1.50	0.20

C) Left perturbations:

	**Sum Sq**	**Mean Sq**	**NumDF**	**DenDF**	**F value**	**Pr(>F)**
**BCI**	0.16	0.16	1.00	751.51	3.42	0.06
**Size**	23.35	11.68	2.00	747.29	243.61	3.50e-82
**Group**	0.02	0.02	1.00	17.28	0.41	0.53
**Marker**	2.31	1.16	2.00	746.81	24.12	7.09e-11
**Age**	0.15	0.15	1.00	17.32	3.17	0.09
**Trial_order**	0.34	0.34	1.00	756.68	7.12	0.01
**Sex**	0.01	0.01	1.00	17.23	0.24	0.63
**Height**	9.43e-04	9.43e-04	1.00	17.49	0.02	0.89
**BCI:Size**	0.19	0.10	2.00	747.70	1.99	0.14
**BCI:Group**	0.04	0.04	1.00	748.95	0.92	0.34
**Size:Group**	0.19	0.09	2.00	747.28	1.94	0.14
**BCI:Marker**	0.08	0.04	2.00	746.58	0.88	0.41

**Size:Marker**	0.15	0.04	4.00	746.49	0.78	0.54
**Group:Marker**	0.02	0.01	2.00	746.81	0.19	0.83
**BCI:Size:Group**	0.46	0.23	2.00	747.53	4.79	0.01
**BCI:Size:Marker**	0.19	0.05	4.00	746.55	0.97	0.42
**BCI:Group:Marker**	0.01	0.01	2.00	746.58	0.14	0.87
**Size:Group:Marker**	0.31	0.08	4.00	746.49	1.64	0.16
**BCI:Size:Group:Marker**	0.09	0.02	4.00	746.55	0.46	0.76

D) Right perturbations:

	**Sum Sq**	**Mean Sq**	**NumDF**	**DenDF**	**F value**	**Pr(>F)**
**BCI**	0.01	0.01	1.00	758.58	0.45	0.50
**Size**	23.58	11.79	2.00	756.29	375.35	5.96e-114
**Group**	0.02	0.02	1.00	17.91	0.73	0.40
**Marker**	2.26	1.13	2.00	756.07	36.05	1.11e-15
**Age**	0.01	0.01	1.00	17.96	0.21	0.65
**Trial_order**	6.49e-04	6.49e-04	1.00	759.46	0.02	0.89
**Sex**	5.12e-04	5.12e-04	1.00	17.92	0.02	0.90
**Height**	0.01	0.01	1.00	18.00	0.20	0.66
**BCI:Size**	0.20	0.10	2.00	756.54	3.11	0.05
**BCI:Group**	0.01	0.01	1.00	757.68	0.33	0.57
**Size:Group**	0.12	0.06	2.00	756.45	1.93	0.15
**BCI:Marker**	0.01	3.24e-03	2.00	756.05	0.10	0.90
**Size:Marker**	0.11	0.03	4.00	756.05	0.88	0.48
**Group:Marker**	0.05	0.02	2.00	756.07	0.74	0.48
**BCI:Size:Group**	0.42	0.21	2.00	756.44	6.73	1.27e-03
**BCI:Size:Marker**	0.10	0.03	4.00	756.06	0.81	0.52
**BCI:Group:Marker**	0.01	4.37e-03	2.00	756.05	0.14	0.87
**Size:Group:Marker**	0.24	0.06	4.00	756.05	1.92	0.11
**BCI:Size:Group:Marker**	0.06	0.01	4.00	756.06	0.45	0.77

Note: Model: Pert.Time_X~BCI*Size*Group*Marker + Age + Trial_order + Sex + Height + (1|`Study.code`). ANOVA using Satterthwaite's method. Degrees-of-freedom adjusted for multiple comparisons using Kenward-Roger method.

Table 7.
Translational P2 latency ANOVA.

A) Forwards perturbations:

	**Sum Sq**	**Mean Sq**	**NumDF**	**DenDF**	**F value**	**Pr(>F)**
**BCI**	0.30	0.30	1.00	1254.00	6.31	0.01
**Size**	40.62	20.31	2.00	1248.63	423.13	5.02e-141
**Group**	0.01	0.01	1.00	25.43	0.14	0.71
**Marker**	16.89	4.22	4.00	1247.90	87.96	6.80e-66
**Age**	1.82e-04	1.82e-04	1.00	17.49	3.80e-03	0.95

**Trial_order**	0.02	0.02	1.00	603.80	0.40	0.53
**Sex**	0.20	0.20	1.00	17.67	4.22	0.05
**Height**	0.06	0.06	1.00	17.61	1.18	0.29
**BCI:Size**	0.59	0.29	2.00	1249.02	6.12	2.27e-03
**BCI:Group**	0.04	0.04	1.00	1253.42	0.75	0.39
**Size:Group**	1.06	0.53	2.00	1248.85	11.07	1.71e-05
**BCI:Marker**	0.47	0.12	4.00	1247.85	2.42	0.05
**Size:Marker**	1.02	0.13	8.00	1247.86	2.65	0.01
**Group:Marker**	0.09	0.02	4.00	1247.90	0.49	0.75
**BCI:Size:Group**	0.94	0.47	2.00	1249.66	9.80	5.97e-05
**BCI:Size:Marker**	0.18	0.02	8.00	1247.85	0.48	0.87
**BCI:Group:Marker**	0.34	0.08	4.00	1247.85	1.75	0.14
**Size:Group:Marker**	0.38	0.05	8.00	1247.86	1.00	0.44
**BCI:Size:Group:Marker**	0.17	0.02	8.00	1247.85	0.43	0.90

B) Backwards perturbations:

	**Sum Sq**	**Mean Sq**	**NumDF**	**DenDF**	**F value**	**Pr(>F)**
**BCI**	0.02	0.02	1.00	1267.71	0.43	0.51
**Size**	29.51	14.75	2.00	1262.84	286.37	2.76e-103
**Group**	0.01	0.01	1.00	20.48	0.16	0.69
**Marker**	19.39	4.85	4.00	1262.85	94.07	4.46e-70
**Age**	0.08	0.08	1.00	16.48	1.54	0.23
**Trial_order**	0.01	0.01	1.00	1008.99	0.10	0.75
**Sex**	0.03	0.03	1.00	16.41	0.58	0.46
**Height**	4.65e-05	4.65e-05	1.00	16.56	9.04e-04	0.98
**BCI:Size**	0.01	4.54e-03	2.00	1263.32	0.09	0.92
**BCI:Group**	0.06	0.06	1.00	1267.05	1.15	0.28
**Size:Group**	1.07	0.53	2.00	1263.62	10.34	3.52e-05
**BCI:Marker**	0.05	0.01	4.00	1262.59	0.24	0.92
**Size:Marker**	0.91	0.11	8.00	1262.57	2.20	0.02
**Group:Marker**	0.30	0.08	4.00	1262.85	1.48	0.21
**BCI:Size:Group**	0.04	0.02	2.00	1263.89	0.42	0.66
**BCI:Size:Marker**	0.19	0.02	8.00	1262.57	0.46	0.88
**BCI:Group:Marker**	4.62e-03	1.15e-03	4.00	1262.59	0.02	1.00
**Size:Group:Marker**	1.03	0.13	8.00	1262.57	2.50	0.01
**BCI:Size:Group:Marker**	0.27	0.03	8.00	1262.57	0.65	0.74

C) Left perturbations:

	**Sum Sq**	**Mean Sq**	**NumDF**	**DenDF**	**F value**	**Pr(>F)**
**BCI**	0.02	0.02	1.00	1277.01	0.19	0.66
**Size**	30.58	15.29	2.00	1273.94	181.03	6.37e-70
**Group**	0.02	0.02	1.00	20.52	0.24	0.63
**Marker**	34.36	8.59	4.00	1272.87	101.70	3.45e-75
**Age**	0.06	0.06	1.00	17.68	0.68	0.42

**Trial_order**	0.11	0.11	1.00	1139.22	1.32	0.25
**Sex**	0.03	0.03	1.00	17.69	0.30	0.59
**Height**	5.26e-04	5.26e-04	1.00	17.72	0.01	0.94
**BCI:Size**	0.23	0.11	2.00	1274.38	1.34	0.26
**BCI:Group**	0.07	0.07	1.00	1276.01	0.88	0.35
**Size:Group**	0.33	0.16	2.00	1273.38	1.94	0.14
**BCI:Marker**	0.16	0.04	4.00	1272.83	0.47	0.75
**Size:Marker**	6.05	0.76	8.00	1272.81	8.95	5.33e-12
**Group:Marker**	1.50	0.37	4.00	1272.87	4.43	1.48e-03
**BCI:Size:Group**	0.39	0.19	2.00	1273.25	2.30	0.10
**BCI:Size:Marker**	0.47	0.06	8.00	1272.82	0.69	0.70
**BCI:Group:Marker**	0.42	0.10	4.00	1272.83	1.24	0.29
**Size:Group:Marker**	0.59	0.07	8.00	1272.81	0.88	0.53
**BCI:Size:Group:Marker**	1.19	0.15	8.00	1272.82	1.76	0.08

D) Right perturbations:

	**Sum Sq**	**Mean Sq**	**NumDF**	**DenDF**	**F value**	**Pr(>F)**
**BCI**	0.63	0.63	1.00	1288.85	7.53	0.01
**Size**	32.01	16.00	2.00	1286.16	190.57	3.27e-73
**Group**	1.93e-03	1.93e-03	1.00	21.63	0.02	0.88
**Marker**	29.66	7.42	4.00	1284.51	88.29	2.47e-66
**Age**	0.02	0.02	1.00	17.23	0.27	0.61
**Trial_order**	0.22	0.22	1.00	1054.46	2.61	0.11
**Sex**	1.70e-03	1.70e-03	1.00	17.32	0.02	0.89
**Height**	1.23e-03	1.23e-03	1.00	17.37	0.01	0.90
**BCI:Size**	0.45	0.23	2.00	1284.93	2.69	0.07
**BCI:Group**	0.08	0.08	1.00	1288.21	0.94	0.33
**Size:Group**	0.35	0.18	2.00	1285.49	2.09	0.12
**BCI:Marker**	0.03	0.01	4.00	1284.50	0.10	0.98
**Size:Marker**	6.20	0.77	8.00	1284.50	9.22	2.04e-12
**Group:Marker**	1.16	0.29	4.00	1284.51	3.44	0.01
**BCI:Size:Group**	0.60	0.30	2.00	1285.18	3.57	0.03
**BCI:Size:Marker**	0.22	0.03	8.00	1284.51	0.32	0.96
**BCI:Group:Marker**	0.06	0.02	4.00	1284.50	0.19	0.94
**Size:Group:Marker**	0.21	0.03	8.00	1284.50	0.32	0.96
**BCI:Size:Group:Marker**	0.58	0.07	8.00	1284.51	0.87	0.54

Note: Model: Pert.Time_X~BCI*Size*Group*Marker + Age + Trial_order + Sex + Height + (1|`Study.code`). ANOVA using Satterthwaite's method. Degrees-of-freedom adjusted for multiple comparisons using Kenward-Roger method.

Table 8.
Rotational P2 latency ANOVA.
A) Forwards perturbations:

	**Sum Sq**	**Mean Sq**	**NumDF**	**DenDF**	**F value**	**Pr(>F)**

**BCI**	0.35	0.35	1.00	739.58	3.17	0.08
**Size**	14.70	7.35	2.00	736.58	65.91	4.64e-27
**Group**	0.01	0.01	1.00	17.75	0.07	0.80
**Marker**	0.63	0.32	2.00	736.19	2.83	0.06
**Age**	0.06	0.06	1.00	17.84	0.53	0.47
**Trial_order**	1.80e-03	1.80e-03	1.00	739.30	0.02	0.90
**Sex**	0.03	0.03	1.00	17.84	0.28	0.60
**Height**	0.16	0.16	1.00	18.05	1.47	0.24
**BCI:Size**	0.46	0.23	2.00	736.46	2.07	0.13
**BCI:Group**	0.35	0.35	1.00	739.04	3.11	0.08
**Size:Group**	0.15	0.08	2.00	736.54	0.69	0.50
**BCI:Marker**	0.08	0.04	2.00	736.07	0.37	0.69
**Size:Marker**	0.61	0.15	4.00	736.09	1.36	0.25
**Group:Marker**	0.02	0.01	2.00	736.19	0.08	0.92
**BCI:Size:Group**	0.13	0.07	2.00	736.44	0.58	0.56
**BCI:Size:Marker**	0.13	0.03	4.00	736.07	0.28	0.89
**BCI:Group:Marker**	0.09	0.04	2.00	736.07	0.39	0.68
**Size:Group:Marker**	0.17	0.04	4.00	736.09	0.38	0.82
**BCI:Size:Group:Marker**	0.12	0.03	4.00	736.07	0.28	0.89

B) Backwards perturbations:

	**Sum Sq**	**Mean Sq**	**NumDF**	**DenDF**	**F value**	**Pr(>F)**
**BCI**	3.52e-03	3.52e-03	1.00	745.02	0.06	0.81
**Size**	4.25	2.12	2.00	742.05	35.47	1.94e-15
**Group**	0.08	0.08	1.00	17.57	1.35	0.26
**Marker**	0.07	0.04	2.00	742.78	0.59	0.55
**Age**	0.07	0.07	1.00	17.74	1.16	0.30
**Trial_order**	0.13	0.13	1.00	745.33	2.12	0.15
**Sex**	0.02	0.02	1.00	17.60	0.32	0.58
**Height**	0.08	0.08	1.00	18.00	1.30	0.27
**BCI:Size**	0.07	0.04	2.00	742.24	0.60	0.55
**BCI:Group**	0.09	0.09	1.00	744.59	1.46	0.23
**Size:Group**	0.04	0.02	2.00	742.05	0.32	0.73
**BCI:Marker**	0.23	0.12	2.00	742.01	1.95	0.14
**Size:Marker**	0.29	0.07	4.00	741.95	1.23	0.30
**Group:Marker**	0.16	0.08	2.00	742.78	1.37	0.25
**BCI:Size:Group**	0.20	0.10	2.00	742.23	1.67	0.19
**BCI:Size:Marker**	0.14	0.03	4.00	741.95	0.58	0.68
**BCI:Group:Marker**	0.09	0.04	2.00	742.01	0.74	0.48
**Size:Group:Marker**	0.06	0.01	4.00	741.95	0.23	0.92
**BCI:Size:Group:Marker**	0.23	0.06	4.00	741.95	0.94	0.44

C) Left perturbations:

	**Sum Sq**	**Mean Sq**	**NumDF**	**DenDF**	**F value**	**Pr(>F)**
**BCI**	0.02	0.02	1.00	750.73	0.30	0.59
**Size**	19.97	9.99	2.00	747.61	161.77	4.17e-59

**Group**	0.03	0.03	1.00	17.91	0.44	0.52
**Marker**	0.60	0.30	2.00	747.29	4.85	0.01
**Age**	4.39e-04	4.39e-04	1.00	17.94	0.01	0.93
**Trial_order**	0.70	0.70	1.00	754.60	11.31	8.08e-04
**Sex**	0.02	0.02	1.00	17.88	0.31	0.59
**Height**	1.93e-04	1.93e-04	1.00	18.07	3.13e-03	0.96
**BCI:Size**	0.11	0.05	2.00	747.90	0.87	0.42
**BCI:Group**	0.01	0.01	1.00	748.84	0.23	0.63
**Size:Group**	0.08	0.04	2.00	747.61	0.63	0.53
**BCI:Marker**	0.09	0.04	2.00	747.13	0.72	0.49
**Size:Marker**	0.06	0.01	4.00	747.08	0.24	0.92
**Group:Marker**	0.01	0.01	2.00	747.29	0.11	0.89
**BCI:Size:Group**	0.15	0.07	2.00	747.78	1.18	0.31
**BCI:Size:Marker**	0.09	0.02	4.00	747.12	0.35	0.84
**BCI:Group:Marker**	0.28	0.14	2.00	747.13	2.25	0.11
**Size:Group:Marker**	0.22	0.06	4.00	747.08	0.90	0.46
**BCI:Size:Group:Marker**	0.11	0.03	4.00	747.12	0.46	0.76

D) Right perturbations:

	**Sum Sq**	**Mean Sq**	**NumDF**	**DenDF**	**F value**	**Pr(>F)**
**BCI**	0.23	0.23	1.00	760.37	3.40	0.07
**Size**	20.90	10.45	2.00	756.38	155.07	3.66e-57
**Group**	0.40	0.40	1.00	17.61	5.88	0.03
**Marker**	0.37	0.18	2.00	755.92	2.72	0.07
**Age**	0.15	0.15	1.00	17.67	2.20	0.16
**Trial_order**	0.03	0.03	1.00	762.33	0.51	0.48
**Sex**	4.73e-04	4.73e-04	1.00	17.61	0.01	0.93
**Height**	0.43	0.43	1.00	17.76	6.33	0.02
**BCI:Size**	0.93	0.47	2.00	756.93	6.90	1.07e-03
**BCI:Group**	0.05	0.05	1.00	758.88	0.71	0.40
**Size:Group**	0.09	0.05	2.00	756.74	0.68	0.51
**BCI:Marker**	0.20	0.10	2.00	755.86	1.47	0.23
**Size:Marker**	0.07	0.02	4.00	755.88	0.27	0.89
**Group:Marker**	0.01	4.03e-03	2.00	755.91	0.06	0.94
**BCI:Size:Group**	0.99	0.50	2.00	756.72	7.36	6.82e-04
**BCI:Size:Marker**	0.05	0.01	4.00	755.89	0.17	0.95
**BCI:Group:Marker**	0.06	0.03	2.00	755.87	0.46	0.63
**Size:Group:Marker**	0.07	0.02	4.00	755.87	0.25	0.91
**BCI:Size:Group:Marker**	0.48	0.12	4.00	755.90	1.79	0.13

Note: Model: Pert.Time_X~BCI*Size*Group*Marker + Age + Trial_order + Sex + Height + (1|`Study.code`). ANOVA using Satterthwaite's method. Degrees-of-freedom adjusted for multiple comparisons using Kenward-Roger method.

Table 9.
Translational inter-peak latency ANOVA.

A) Forwards perturbations:

	Sum Sq	Mean Sq	NumDF	DenDF	F value	Pr(>F)
**BCI**	0.89	0.89	1.00	1251.78	15.98	6.77e-05
**Size**	1.37	0.68	2.00	1248.25	12.31	5.09e-06
**Group**	0.04	0.04	1.00	22.69	0.67	0.42
**Marker**	5.34	1.33	4.00	1247.84	24.00	3.76e-19
**Age**	0.06	0.06	1.00	17.59	1.13	0.30
**Trial_order**	0.13	0.13	1.00	942.11	2.42	0.12
**Sex**	3.59e-03	3.59e-03	1.00	17.71	0.06	0.80
**Height**	0.12	0.12	1.00	17.66	2.11	0.16
**BCI:Size**	0.84	0.42	2.00	1248.51	7.56	5.46e-04
**BCI:Group**	2.38e-03	2.38e-03	1.00	1251.36	0.04	0.84
**Size:Group**	0.38	0.19	2.00	1248.41	3.40	0.03
**BCI:Marker**	0.40	0.10	4.00	1247.81	1.79	0.13
**Size:Marker**	1.17	0.15	8.00	1247.81	2.64	0.01
**Group:Marker**	0.57	0.14	4.00	1247.84	2.54	0.04
**BCI:Size:Group**	1.05	0.52	2.00	1248.93	9.40	8.86e-05
**BCI:Size:Marker**	0.17	0.02	8.00	1247.80	0.39	0.93
**BCI:Group:Marker**	0.65	0.16	4.00	1247.81	2.92	0.02
**Size:Group:Marker**	0.21	0.03	8.00	1247.81	0.46	0.88
**BCI:Size:Group:Marker**	0.17	0.02	8.00	1247.80	0.38	0.93

B) Backwards perturbations:

	Sum Sq	Mean Sq	NumDF	DenDF	F value	Pr(>F)
**BCI**	0.56	0.56	1.00	1271.90	8.69	3.27e-03
**Size**	6.16	3.08	2.00	1263.27	48.06	7.68e-21
**Group**	1.07	1.07	1.00	23.94	16.68	4.28e-04
**Marker**	2.86	0.71	4.00	1263.30	11.15	6.80e-09
**Age**	0.28	0.28	1.00	16.38	4.39	0.05
**Trial_order**	0.16	0.16	1.00	515.89	2.54	0.11
**Sex**	8.00e-04	8.00e-04	1.00	16.22	0.01	0.91
**Height**	0.08	0.08	1.00	16.56	1.31	0.27
**BCI:Size**	1.36	0.68	2.00	1264.28	10.58	2.76e-05
**BCI:Group**	0.38	0.38	1.00	1270.89	5.90	0.02
**Size:Group**	1.07	0.53	2.00	1264.73	8.34	2.52e-04
**BCI:Marker**	0.15	0.04	4.00	1262.76	0.59	0.67
**Size:Marker**	2.19	0.27	8.00	1262.71	4.28	4.37e-05
**Group:Marker**	1.67	0.42	4.00	1263.30	6.52	3.42e-05
**BCI:Size:Group**	0.70	0.35	2.00	1265.32	5.43	4.47e-03
**BCI:Size:Marker**	0.35	0.04	8.00	1262.71	0.68	0.71
**BCI:Group:Marker**	0.20	0.05	4.00	1262.76	0.79	0.53
**Size:Group:Marker**	0.45	0.06	8.00	1262.71	0.88	0.53
**BCI:Size:Group:Marker**	0.27	0.03	8.00	1262.71	0.53	0.84

C) Left perturbations:

	**Sum Sq**	**Mean Sq**	**NumDF**	**DenDF**	**F value**	**Pr(>F)**
**BCI**	0.22	0.22	1.00	1280.57	3.02	0.08
**Size**	0.15	0.07	2.00	1274.11	1.04	0.36
**Group**	0.67	0.67	1.00	17.94	9.35	0.01
**Marker**	16.85	4.21	4.00	1273.34	59.17	7.36e-46
**Age**	0.32	0.32	1.00	18.09	4.55	0.05
**Trial_order**	1.57	1.57	1.00	1287.97	22.10	2.87e-06
**Sex**	4.81e-04	4.81e-04	1.00	17.96	0.01	0.94
**Height**	0.02	0.02	1.00	18.21	0.24	0.63
**BCI:Size**	0.74	0.37	2.00	1274.57	5.21	0.01
**BCI:Group**	0.14	0.14	1.00	1277.00	1.97	0.16
**Size:Group**	0.47	0.23	2.00	1274.10	3.29	0.04
**BCI:Marker**	0.11	0.03	4.00	1273.24	0.39	0.81
**Size:Marker**	0.39	0.05	8.00	1273.19	0.68	0.71
**Group:Marker**	1.79	0.45	4.00	1273.34	6.30	5.06e-05
**BCI:Size:Group**	1.45	0.72	2.00	1274.37	10.18	4.10e-05
**BCI:Size:Marker**	0.65	0.08	8.00	1273.20	1.13	0.34
**BCI:Group:Marker**	0.17	0.04	4.00	1273.24	0.60	0.66
**Size:Group:Marker**	0.94	0.12	8.00	1273.19	1.65	0.11
**BCI:Size:Group:Marker**	0.32	0.04	8.00	1273.20	0.56	0.81

D) Right perturbations:

	**Sum Sq**	**Mean Sq**	**NumDF**	**DenDF**	**F value**	**Pr(>F)**
**BCI**	0.02	0.02	1.00	1290.31	0.38	0.54
**Size**	0.31	0.16	2.00	1287.21	2.64	0.07
**Group**	0.12	0.12	1.00	23.53	1.96	0.17
**Marker**	12.81	3.20	4.00	1285.27	53.98	4.25e-42
**Age**	0.08	0.08	1.00	17.89	1.32	0.26
**Trial_order**	4.85e-03	4.85e-03	1.00	939.54	0.08	0.78
**Sex**	0.03	0.03	1.00	18.02	0.56	0.46
**Height**	0.01	0.01	1.00	18.08	0.22	0.65
**BCI:Size**	0.13	0.06	2.00	1285.79	1.06	0.35
**BCI:Group**	0.03	0.03	1.00	1289.61	0.50	0.48
**Size:Group**	0.59	0.30	2.00	1286.43	4.99	0.01
**BCI:Marker**	0.10	0.03	4.00	1285.27	0.44	0.78
**Size:Marker**	0.44	0.06	8.00	1285.27	0.93	0.49
**Group:Marker**	0.72	0.18	4.00	1285.27	3.05	0.02
**BCI:Size:Group**	0.57	0.28	2.00	1286.08	4.80	0.01
**BCI:Size:Marker**	0.22	0.03	8.00	1285.27	0.47	0.88
**BCI:Group:Marker**	0.24	0.06	4.00	1285.27	1.03	0.39
**Size:Group:Marker**	0.22	0.03	8.00	1285.27	0.46	0.89

BCI:Size:Group:Marker	0.14	0.02	8.00	1285.27	0.29	0.97

Note: Model: value~BCI*Size*Group*Marker + Age + Trial_order + Sex + Height + (1|`Study.code`). ANOVA using Satterthwaite's method. Degrees-of-freedom adjusted for multiple comparisons using Kenward-Roger method.

Table 10.
Rotational inter-peak latency ANOVA.
A) Forwards perturbations:

	Sum Sq	Mean Sq	NumDF	DenDF	F value	Pr(>F)
**BCI**	0.37	0.37	1.00	740.30	3.01	0.08
**Size**	3.03	1.52	2.00	736.36	12.39	5.12e-06
**Group**	0.75	0.75	1.00	17.17	6.15	0.02
**Marker**	8.81	4.41	2.00	735.78	35.99	1.23e-15
**Age**	0.23	0.23	1.00	17.28	1.84	0.19
**Trial_order**	0.62	0.62	1.00	740.27	5.05	0.02
**Sex**	0.04	0.04	1.00	17.29	0.29	0.60
**Height**	3.79e-03	3.79e-03	1.00	17.58	0.03	0.86
**BCI:Size**	0.09	0.04	2.00	736.19	0.36	0.69
**BCI:Group**	0.01	0.01	1.00	739.60	0.11	0.74
**Size:Group**	0.93	0.47	2.00	736.30	3.80	0.02
**BCI:Marker**	0.05	0.02	2.00	735.60	0.19	0.83
**Size:Marker**	0.78	0.19	4.00	735.63	1.58	0.18
**Group:Marker**	1.43	0.72	2.00	735.78	5.86	2.99e-03
**BCI:Size:Group**	0.35	0.17	2.00	736.15	1.41	0.24
**BCI:Size:Marker**	0.27	0.07	4.00	735.60	0.55	0.70
**BCI:Group:Marker**	0.20	0.10	2.00	735.60	0.81	0.44
**Size:Group:Marker**	0.44	0.11	4.00	735.63	0.90	0.46
**BCI:Size:Group:Marker**	0.09	0.02	4.00	735.60	0.18	0.95

B) Backwards perturbations:

	Sum Sq	Mean Sq	NumDF	DenDF	F value	Pr(>F)
**BCI**	0.01	0.01	1.00	746.28	0.14	0.71
**Size**	3.45	1.73	2.00	742.25	21.21	1.10e-09
**Group**	0.30	0.30	1.00	17.52	3.64	0.07
**Marker**	4.08	2.04	2.00	743.30	25.06	2.94e-11
**Age**	0.01	0.01	1.00	17.78	0.14	0.71
**Trial_order**	0.56	0.56	1.00	746.95	6.90	0.01
**Sex**	0.02	0.02	1.00	17.57	0.19	0.66
**Height**	0.13	0.13	1.00	18.17	1.60	0.22
**BCI:Size**	0.29	0.15	2.00	742.54	1.79	0.17
**BCI:Group**	8.30e-05	8.30e-05	1.00	745.72	1.02e-03	0.97
**Size:Group**	0.21	0.11	2.00	742.25	1.32	0.27
**BCI:Marker**	0.50	0.25	2.00	742.19	3.07	0.05
**Size:Marker**	0.10	0.02	4.00	742.09	0.30	0.88

**Group:Marker**	0.83	0.41	2.00	743.30	5.09	0.01
**BCI:Size:Group**	0.15	0.08	2.00	742.53	0.95	0.39
**BCI:Size:Marker**	0.34	0.08	4.00	742.09	1.03	0.39
**BCI:Group:Marker**	0.10	0.05	2.00	742.19	0.60	0.55
**Size:Group:Marker**	0.16	0.04	4.00	742.09	0.48	0.75
**BCI:Size:Group:Marker**	0.46	0.11	4.00	742.09	1.41	0.23

C) Left perturbations:

	**Sum Sq**	**Mean Sq**	**NumDF**	**DenDF**	**F value**	**Pr(>F)**
**BCI**	0.30	0.30	1.00	750.12	4.60	0.03
**Size**	0.42	0.21	2.00	747.36	3.18	0.04
**Group**	1.84e-03	1.84e-03	1.00	17.77	0.03	0.87
**Marker**	0.63	0.32	2.00	747.10	4.83	0.01
**Age**	0.08	0.08	1.00	17.80	1.16	0.30
**Trial_order**	0.06	0.06	1.00	753.54	0.87	0.35
**Sex**	0.04	0.04	1.00	17.74	0.65	0.43
**Height**	1.05e-03	1.05e-03	1.00	17.90	0.02	0.90
**BCI:Size**	0.02	0.01	2.00	747.61	0.16	0.85
**BCI:Group**	0.01	0.01	1.00	748.45	0.11	0.74
**Size:Group**	0.19	0.09	2.00	747.36	1.44	0.24
**BCI:Marker**	0.05	0.02	2.00	746.96	0.38	0.69
**Size:Marker**	0.04	0.01	4.00	746.91	0.17	0.95
**Group:Marker**	4.37e-03	2.19e-03	2.00	747.10	0.03	0.97
**BCI:Size:Group**	0.11	0.06	2.00	747.51	0.85	0.43
**BCI:Size:Marker**	0.34	0.09	4.00	746.94	1.30	0.27
**BCI:Group:Marker**	0.22	0.11	2.00	746.96	1.71	0.18
**Size:Group:Marker**	0.04	0.01	4.00	746.91	0.17	0.95
**BCI:Size:Group:Marker**	0.24	0.06	4.00	746.94	0.93	0.45

D) Right perturbations:

	**Sum Sq**	**Mean Sq**	**NumDF**	**DenDF**	**F value**	**Pr(>F)**
**BCI**	0.13	0.13	1.00	760.38	1.90	0.17
**Size**	0.17	0.08	2.00	755.97	1.19	0.30
**Group**	0.82	0.82	1.00	17.12	11.74	3.19e-03
**Marker**	0.82	0.41	2.00	755.44	5.88	2.92e-03
**Age**	0.28	0.28	1.00	17.18	4.02	0.06
**Trial_order**	0.02	0.02	1.00	762.66	0.29	0.59
**Sex**	8.59e-05	8.59e-05	1.00	17.11	1.22e-03	0.97
**Height**	0.33	0.33	1.00	17.27	4.72	0.04
**BCI:Size**	0.40	0.20	2.00	756.61	2.82	0.06
**BCI:Group**	0.01	0.01	1.00	758.75	0.20	0.65
**Size:Group**	0.22	0.11	2.00	756.39	1.54	0.21
**BCI:Marker**	0.24	0.12	2.00	755.37	1.74	0.18
**Size:Marker**	0.08	0.02	4.00	755.38	0.29	0.88
**Group:Marker**	0.04	0.02	2.00	755.42	0.26	0.77

**BCI:Size:Group**	0.18	0.09	2.00	756.37	1.26	0.28
**BCI:Size:Marker**	0.02	4.99e-03	4.00	755.41	0.07	0.99
**BCI:Group:Marker**	0.12	0.06	2.00	755.38	0.83	0.44
**Size:Group:Marker**	0.37	0.09	4.00	755.38	1.31	0.26
**BCI:Size:Group:Marker**	0.45	0.11	4.00	755.41	1.61	0.17

Note: Model: value~BCI*Size*Group*Marker + Age + Trial_order + Sex + Height + (1|`Study.code`). ANOVA using Satterthwaite's method. Degrees-of-freedom adjusted for multiple comparisons using Kenward-Roger method.

Table 11.
Translational P1 amplitudes ANOVA.

A) Forwards perturbations:

	**Sum Sq**	**Mean Sq**	**NumDF**	**DenDF**	**F value**	**Pr(>F)**
**BCI**	37.15	37.15	1.00	1250.89	0.58	0.45
**Size**	81695.18	40847.59	2.00	1248.42	636.05	3.42e-191
**Group**	166.32	166.32	1.00	17.96	2.59	0.12
**Marker**	10324.08	2581.02	4.00	1248.11	40.19	1.05e-31
**Age**	301.62	301.62	1.00	18.01	4.70	0.04
**Trial_order**	267.85	267.85	1.00	1250.36	4.17	0.04
**Sex**	143.71	143.71	1.00	18.01	2.24	0.15
**Height**	260.56	260.56	1.00	18.09	4.06	0.06
**BCI:Size**	782.09	391.04	2.00	1248.38	6.09	2.34e-03
**BCI:Group**	31.07	31.07	1.00	1250.43	0.48	0.49
**Size:Group**	2004.48	1002.24	2.00	1248.40	15.61	2.02e-07
**BCI:Marker**	601.63	150.41	4.00	1248.08	2.34	0.05
**Size:Marker**	1820.62	227.58	8.00	1248.09	3.54	4.56e-04
**Group:Marker**	2031.85	507.96	4.00	1248.11	7.91	2.70e-06
**BCI:Size:Group**	315.71	157.86	2.00	1248.35	2.46	0.09
**BCI:Size:Marker**	142.45	17.81	8.00	1248.08	0.28	0.97
**BCI:Group:Marker**	344.78	86.20	4.00	1248.08	1.34	0.25
**Size:Group:Marker**	876.75	109.59	8.00	1248.09	1.71	0.09
**BCI:Size:Group:Marker**	54.99	6.87	8.00	1248.08	0.11	1.00

B) Backwards perturbations:

	**Sum Sq**	**Mean Sq**	**NumDF**	**DenDF**	**F value**	**Pr(>F)**
**BCI**	449.36	449.36	1.00	1267.35	6.76	0.01
**Size**	133821.70	66910.85	2.00	1263.94	1006.14	3.87e-262
**Group**	1554.48	1554.48	1.00	17.59	23.37	1.41e-04
**Marker**	43379.59	10844.90	4.00	1264.09	163.07	1.32e-112
**Age**	58.55	58.55	1.00	17.73	0.88	0.36
**Trial_order**	203.59	203.59	1.00	1267.76	3.06	0.08
**Sex**	52.87	52.87	1.00	17.63	0.79	0.38

**Height**	1.03	1.03	1.00	17.84	0.02	0.90
**BCI:Size**	909.26	454.63	2.00	1264.15	6.84	1.11e-03
**BCI:Group**	0.30	0.30	1.00	1266.81	4.51e-03	0.95
**Size:Group**	6774.93	3387.47	2.00	1263.93	50.94	5.30e-22
**BCI:Marker**	131.61	32.90	4.00	1263.83	0.49	0.74
**Size:Marker**	10231.71	1278.96	8.00	1263.81	19.23	1.68e-27
**Group:Marker**	1044.14	261.04	4.00	1264.09	3.93	3.58e-03
**BCI:Size:Group**	125.07	62.54	2.00	1264.15	0.94	0.39
**BCI:Size:Marker**	150.42	18.80	8.00	1263.81	0.28	0.97
**BCI:Group:Marker**	122.77	30.69	4.00	1263.83	0.46	0.76
**Size:Group:Marker**	553.01	69.13	8.00	1263.81	1.04	0.40
**BCI:Size:Group:Marker**	147.96	18.49	8.00	1263.81	0.28	0.97

C) Left perturbations:

	**Sum Sq**	**Mean Sq**	**NumDF**	**DenDF**	**F value**	**Pr(>F)**
**BCI**	49.54	49.54	1.00	1274.71	1.29	0.26
**Size**	26501.92	13250.96	2.00	1273.19	343.85	4.00e-120
**Group**	119.84	119.84	1.00	17.97	3.11	0.09
**Marker**	17035.66	4258.92	4.00	1273.05	110.51	6.65e-81
**Age**	8.88	8.88	1.00	18.01	0.23	0.64
**Trial_order**	120.43	120.43	1.00	1276.91	3.12	0.08
**Sex**	133.63	133.63	1.00	17.98	3.47	0.08
**Height**	29.79	29.79	1.00	18.03	0.77	0.39
**BCI:Size**	387.92	193.96	2.00	1273.28	5.03	0.01
**BCI:Group**	568.51	568.51	1.00	1273.84	14.75	1.29e-04
**Size:Group**	1547.55	773.77	2.00	1273.19	20.08	2.60e-09
**BCI:Marker**	13.36	3.34	4.00	1273.03	0.09	0.99
**Size:Marker**	4543.55	567.94	8.00	1273.02	14.74	9.12e-21
**Group:Marker**	47.49	11.87	4.00	1273.05	0.31	0.87
**BCI:Size:Group**	2386.10	1193.05	2.00	1273.24	30.96	7.44e-14
**BCI:Size:Marker**	94.72	11.84	8.00	1273.02	0.31	0.96
**BCI:Group:Marker**	8.45	2.11	4.00	1273.03	0.05	0.99
**Size:Group:Marker**	452.03	56.50	8.00	1273.02	1.47	0.16
**BCI:Size:Group:Marker**	24.84	3.11	8.00	1273.02	0.08	1.00

D) Right perturbations:

	**Sum Sq**	**Mean Sq**	**NumDF**	**DenDF**	**F value**	**Pr(>F)**
**BCI**	146.32	146.32	1.00	1286.11	4.24	0.04
**Size**	23874.57	11937.29	2.00	1285.08	345.73	7.24e-121
**Group**	109.41	109.41	1.00	17.93	3.17	0.09

**Marker**	20141.44	5035.36	4.00	1285.00	145.84	7.30e-103
**Age**	0.40	0.40	1.00	17.94	0.01	0.92
**Trial_order**	0.74	0.74	1.00	1286.63	0.02	0.88
**Sex**	32.18	32.18	1.00	17.97	0.93	0.35
**Height**	3.01	3.01	1.00	17.99	0.09	0.77
**BCI:Size**	224.27	112.13	2.00	1285.13	3.25	0.04
**BCI:Group**	485.27	485.27	1.00	1285.88	14.05	1.85e-04
**Size:Group**	1939.17	969.58	2.00	1285.13	28.08	1.16e-12
**BCI:Marker**	20.64	5.16	4.00	1284.99	0.15	0.96
**Size:Marker**	5132.40	641.55	8.00	1284.99	18.58	1.48e-26
**Group:Marker**	159.48	39.87	4.00	1285.00	1.15	0.33
**BCI:Size:Group**	1122.56	561.28	2.00	1285.13	16.26	1.07e-07
**BCI:Size:Marker**	433.01	54.13	8.00	1285.00	1.57	0.13
**BCI:Group:Marker**	84.02	21.01	4.00	1284.99	0.61	0.66
**Size:Group:Marker**	702.01	87.75	8.00	1284.99	2.54	0.01
**BCI:Size:Group:Marker**	272.51	34.06	8.00	1285.00	0.99	0.44

Note: Model: value~BCI*Size*Group*Marker + Age + Trial_order + Sex + Height + (1|`Study.code`). ANOVA using Satterthwaite's method. Degrees-of-freedom adjusted for multiple comparisons using Kenward-Roger method.

Table 12.
Rotational P1 amplitudes ANOVA.
A) Forwards perturbations:

	**Sum Sq**	**Mean Sq**	**NumDF**	**DenDF**	**F value**	**Pr(>F)**
**BCI**	7.90	7.90	1.00	737.01	0.35	0.55
**Size**	2176.07	1088.04	2.00	736.14	48.85	1.20e-20
**Group**	11.12	11.12	1.00	17.94	0.50	0.49
**Marker**	1429.65	714.83	2.00	736.05	32.09	4.32e-14
**Age**	0.10	0.10	1.00	17.97	4.41e-03	0.95
**Trial_order**	26.61	26.61	1.00	736.80	1.19	0.27
**Sex**	30.04	30.04	1.00	17.97	1.35	0.26
**Height**	1.43	1.43	1.00	18.02	0.06	0.80
**BCI:Size**	73.48	36.74	2.00	736.11	1.65	0.19
**BCI:Group**	183.99	183.99	1.00	736.84	8.26	4.17e-03
**Size:Group**	184.06	92.03	2.00	736.13	4.13	0.02
**BCI:Marker**	49.98	24.99	2.00	736.02	1.12	0.33
**Size:Marker**	328.60	82.15	4.00	736.03	3.69	0.01
**Group:Marker**	188.79	94.40	2.00	736.05	4.24	0.01
**BCI:Size:Group**	51.00	25.50	2.00	736.11	1.14	0.32
**BCI:Size:Marker**	71.03	17.76	4.00	736.02	0.80	0.53
**BCI:Group:Marker**	120.98	60.49	2.00	736.02	2.72	0.07
**Size:Group:Marker**	52.04	13.01	4.00	736.03	0.58	0.67
**BCI:Size:Group:Marker**	73.87	18.47	4.00	736.02	0.83	0.51

B) Backwards perturbations:

	Sum Sq	Mean Sq	NumDF	DenDF	F value	Pr(>F)
**BCI**	0.03	0.03	1.00	745.38	0.60	0.44
**Size**	13.94	6.97	2.00	741.58	156.73	1.69e-57
**Group**	0.03	0.03	1.00	16.96	0.76	0.39
**Marker**	5.11	2.56	2.00	742.54	57.51	6.02e-24
**Age**	0.04	0.04	1.00	17.18	0.95	0.34
**Trial_order**	1.23	1.23	1.00	745.93	27.57	1.98e-07
**Sex**	0.08	0.08	1.00	17.00	1.75	0.20
**Height**	2.51e-04	2.51e-04	1.00	17.52	0.01	0.94
**BCI:Size**	0.32	0.16	2.00	741.84	3.64	0.03
**BCI:Group**	0.09	0.09	1.00	744.85	1.92	0.17
**Size:Group**	0.43	0.22	2.00	741.58	4.84	0.01
**BCI:Marker**	0.12	0.06	2.00	741.52	1.31	0.27
**Size:Marker**	0.12	0.03	4.00	741.43	0.70	0.59
**Group:Marker**	0.40	0.20	2.00	742.54	4.48	0.01
**BCI:Size:Group**	0.45	0.22	2.00	741.83	5.03	0.01
**BCI:Size:Marker**	0.18	0.05	4.00	741.43	1.03	0.39
**BCI:Group:Marker**	0.04	0.02	2.00	741.52	0.47	0.62
**Size:Group:Marker**	0.09	0.02	4.00	741.43	0.53	0.72
**BCI:Size:Group:Marker**	0.27	0.07	4.00	741.43	1.50	0.20

C) Left perturbations:

	Sum Sq	Mean Sq	NumDF	DenDF	F value	Pr(>F)
**BCI**	40.13	40.13	1.00	748.77	2.53	0.11
**Size**	4957.32	2478.66	2.00	747.17	155.99	2.45e-57
**Group**	0.25	0.25	1.00	17.85	0.02	0.90
**Marker**	790.61	395.31	2.00	747.03	24.88	3.47e-11
**Age**	0.39	0.39	1.00	17.87	0.02	0.88
**Trial_order**	231.04	231.04	1.00	750.78	14.54	1.49e-04
**Sex**	6.45	6.45	1.00	17.83	0.41	0.53
**Height**	5.76	5.76	1.00	17.92	0.36	0.55
**BCI:Size**	222.19	111.10	2.00	747.31	6.99	9.81e-04
**BCI:Group**	96.08	96.08	1.00	747.80	6.05	0.01
**Size:Group**	0.74	0.37	2.00	747.17	0.02	0.98
**BCI:Marker**	39.65	19.82	2.00	746.95	1.25	0.29
**Size:Marker**	141.41	35.35	4.00	746.92	2.22	0.06
**Group:Marker**	62.88	31.44	2.00	747.03	1.98	0.14
**BCI:Size:Group**	94.83	47.42	2.00	747.25	2.98	0.05
**BCI:Size:Marker**	36.92	9.23	4.00	746.94	0.58	0.68
**BCI:Group:Marker**	1.67	0.84	2.00	746.95	0.05	0.95
**Size:Group:Marker**	74.51	18.63	4.00	746.92	1.17	0.32
**BCI:Size:Group:Marker**	7.04	1.76	4.00	746.94	0.11	0.98

D) Right perturbations:

	Sum Sq	Mean Sq	NumDF	DenDF	F value	Pr(>F)
**BCI**	7.94	7.94	1.00	757.16	0.41	0.52
**Size**	6330.00	3165.00	2.00	756.13	161.86	3.08e-59
**Group**	13.92	13.92	1.00	17.97	0.71	0.41
**Marker**	583.66	291.83	2.00	756.04	14.92	4.40e-07
**Age**	4.19	4.19	1.00	17.99	0.21	0.65
**Trial_order**	1.67	1.67	1.00	757.50	0.09	0.77
**Sex**	41.62	41.62	1.00	17.97	2.13	0.16
**Height**	75.61	75.61	1.00	18.01	3.87	0.06
**BCI:Size**	15.53	7.77	2.00	756.23	0.40	0.67
**BCI:Group**	137.52	137.52	1.00	756.74	7.03	0.01
**Size:Group**	62.15	31.07	2.00	756.19	1.59	0.20
**BCI:Marker**	5.00	2.50	2.00	756.03	0.13	0.88
**Size:Marker**	149.16	37.29	4.00	756.03	1.91	0.11
**Group:Marker**	83.49	41.74	2.00	756.04	2.13	0.12
**BCI:Size:Group**	237.13	118.57	2.00	756.18	6.06	2.44e-03
**BCI:Size:Marker**	12.04	3.01	4.00	756.03	0.15	0.96
**BCI:Group:Marker**	2.24	1.12	2.00	756.03	0.06	0.94
**Size:Group:Marker**	15.19	3.80	4.00	756.03	0.19	0.94
**BCI:Size:Group:Marker**	22.39	5.60	4.00	756.03	0.29	0.89

Note: Model: value~BCI*Size*Group*Marker + Age + Trial_order + Sex + Height + (1|`Study.code`). ANOVA using Satterthwaite's method. Degrees-of-freedom adjusted for multiple comparisons using Kenward-Roger method.

Table 13.
Translational P2 amplitudes ANOVA.
A) Forwards perturbations:

	Sum Sq	Mean Sq	NumDF	DenDF	F value	Pr(>F)
**BCI**	212.82	212.82	1.00	1250.17	2.10	0.15
**Size**	60201.71	30100.86	2.00	1248.32	297.35	2.46e-106
**Group**	77.94	77.94	1.00	17.99	0.77	0.39
**Marker**	4039.93	1009.98	4.00	1248.09	9.98	6.00e-08
**Age**	490.36	490.36	1.00	18.02	4.84	0.04
**Trial_order**	3922.56	3922.56	1.00	1249.74	38.75	6.57e-10
**Sex**	113.48	113.48	1.00	18.02	1.12	0.30
**Height**	328.46	328.46	1.00	18.08	3.24	0.09
**BCI:Size**	1600.38	800.19	2.00	1248.29	7.90	3.88e-04
**BCI:Group**	328.05	328.05	1.00	1249.83	3.24	0.07
**Size:Group**	4927.64	2463.82	2.00	1248.30	24.34	4.27e-11
**BCI:Marker**	1077.65	269.41	4.00	1248.07	2.66	0.03
**Size:Marker**	1136.51	142.06	8.00	1248.08	1.40	0.19
**Group:Marker**	856.43	214.11	4.00	1248.09	2.12	0.08
**BCI:Size:Group**	890.94	445.47	2.00	1248.26	4.40	0.01

**BCI:Size:Marker**	300.81	37.60	8.00	1248.07	0.37	0.94
**BCI:Group:Marker**	357.55	89.39	4.00	1248.07	0.88	0.47
**Size:Group:Marker**	495.07	61.88	8.00	1248.08	0.61	0.77
**BCI:Size:Group:Marker**	50.62	6.33	8.00	1248.07	0.06	1.00

B) Backwards perturbations:

	**Sum Sq**	**Mean Sq**	**NumDF**	**DenDF**	**F value**	**Pr(>F)**
**BCI**	1855.28	1855.28	1.00	1266.84	21.67	3.58e-06
**Size**	107207.48	53603.74	2.00	1263.89	626.15	1.06e-189
**Group**	2469.41	2469.41	1.00	17.60	28.85	4.52e-05
**Marker**	5355.40	1338.85	4.00	1264.03	15.64	1.70e-12
**Age**	153.77	153.77	1.00	17.72	1.80	0.20
**Trial_order**	2418.86	2418.86	1.00	1267.17	28.25	1.26e-07
**Sex**	165.83	165.83	1.00	17.64	1.94	0.18
**Height**	1.61	1.61	1.00	17.82	0.02	0.89
**BCI:Size**	1628.19	814.09	2.00	1264.07	9.51	7.96e-05
**BCI:Group**	274.75	274.75	1.00	1266.37	3.21	0.07
**Size:Group**	9196.96	4598.48	2.00	1263.89	53.72	4.08e-23
**BCI:Marker**	200.04	50.01	4.00	1263.81	0.58	0.67
**Size:Marker**	6214.59	776.82	8.00	1263.79	9.07	3.48e-12
**Group:Marker**	1206.06	301.52	4.00	1264.03	3.52	0.01
**BCI:Size:Group**	728.43	364.22	2.00	1264.07	4.25	0.01
**BCI:Size:Marker**	168.79	21.10	8.00	1263.79	0.25	0.98
**BCI:Group:Marker**	123.37	30.84	4.00	1263.81	0.36	0.84
**Size:Group:Marker**	419.04	52.38	8.00	1263.79	0.61	0.77
**BCI:Size:Group:Marker**	358.61	44.83	8.00	1263.79	0.52	0.84

C) Left perturbations:

	**Sum Sq**	**Mean Sq**	**NumDF**	**DenDF**	**F value**	**Pr(>F)**
**BCI**	288.11	288.11	1.00	1274.66	5.28	0.02
**Size**	14702.68	7351.34	2.00	1273.19	134.79	7.99e-54
**Group**	100.22	100.22	1.00	17.99	1.84	0.19
**Marker**	1322.50	330.62	4.00	1273.06	6.06	7.84e-05
**Age**	48.90	48.90	1.00	18.02	0.90	0.36
**Trial_order**	582.45	582.45	1.00	1276.78	10.68	1.11e-03
**Sex**	201.66	201.66	1.00	17.99	3.70	0.07
**Height**	147.69	147.69	1.00	18.04	2.71	0.12
**BCI:Size**	417.14	208.57	2.00	1273.28	3.82	0.02

**BCI:Group**	652.06	652.06	1.00	1273.82	11.96	5.63e-04
**Size:Group**	3243.55	1621.78	2.00	1273.19	29.74	2.39e-13
**BCI:Marker**	103.71	25.93	4.00	1273.04	0.48	0.75
**Size:Marker**	1280.13	160.02	8.00	1273.03	2.93	2.99e-03
**Group:Marker**	123.65	30.91	4.00	1273.06	0.57	0.69
**BCI:Size:Group**	3930.48	1965.24	2.00	1273.24	36.03	5.99e-16
**BCI:Size:Marker**	243.62	30.45	8.00	1273.03	0.56	0.81
**BCI:Group:Marker**	95.97	23.99	4.00	1273.04	0.44	0.78
**Size:Group:Marker**	746.15	93.27	8.00	1273.03	1.71	0.09
**BCI:Size:Group:Marker**	26.09	3.26	8.00	1273.03	0.06	1.00

D) Right perturbations:

	**Sum Sq**	**Mean Sq**	**NumDF**	**DenDF**	**F value**	**Pr(>F)**
**BCI**	342.29	342.29	1.00	1286.33	6.29	0.01
**Size**	8500.02	4250.01	2.00	1285.09	78.04	1.03e-32
**Group**	174.00	174.00	1.00	17.91	3.20	0.09
**Marker**	1107.19	276.80	4.00	1284.99	5.08	4.59e-04
**Age**	1.13	1.13	1.00	17.92	0.02	0.89
**Trial_order**	120.91	120.91	1.00	1286.96	2.22	0.14
**Sex**	20.94	20.94	1.00	17.95	0.38	0.54
**Height**	5.73	5.73	1.00	17.98	0.11	0.75
**BCI:Size**	285.02	142.51	2.00	1285.15	2.62	0.07
**BCI:Group**	935.93	935.93	1.00	1286.06	17.19	3.61e-05
**Size:Group**	3042.25	1521.12	2.00	1285.15	27.93	1.34e-12
**BCI:Marker**	33.54	8.38	4.00	1284.98	0.15	0.96
**Size:Marker**	1390.44	173.80	8.00	1284.98	3.19	1.36e-03
**Group:Marker**	135.53	33.88	4.00	1284.99	0.62	0.65
**BCI:Size:Group**	1789.20	894.60	2.00	1285.15	16.43	9.03e-08
**BCI:Size:Marker**	584.62	73.08	8.00	1284.98	1.34	0.22
**BCI:Group:Marker**	140.85	35.21	4.00	1284.98	0.65	0.63
**Size:Group:Marker**	772.05	96.51	8.00	1284.98	1.77	0.08
**BCI:Size:Group:Marker**	583.49	72.94	8.00	1284.98	1.34	0.22

Note: Model: value~BCI*Size*Group*Marker + Age + Trial_order + Sex + Height + (1|`Study.code`). ANOVA using Satterthwaite's method. Degrees-of-freedom adjusted for multiple comparisons using Kenward-Roger method.

Table 14.

Rotational P2 amplitudes ANOVA.

A) Forwards perturbations:

	**Sum Sq**	**Mean Sq**	**NumDF**	**DenDF**	**F value**	**Pr(>F)**
**BCI**	116.51	116.51	1.00	737.47	1.79	0.18
**Size**	1776.26	888.13	2.00	736.16	13.68	1.46e-06
**Group**	386.38	386.38	1.00	17.85	5.95	0.03
**Marker**	7330.19	3665.09	2.00	736.02	56.46	1.54e-23

**Age**	22.34	22.34	1.00	17.89	0.34	0.56
**Trial_order**	1759.20	1759.20	1.00	737.19	27.10	2.51e-07
**Sex**	0.96	0.96	1.00	17.89	0.01	0.90
**Height**	153.97	153.97	1.00	17.97	2.37	0.14
**BCI:Size**	329.12	164.56	2.00	736.12	2.54	0.08
**BCI:Group**	139.76	139.76	1.00	737.23	2.15	0.14
**Size:Group**	568.29	284.14	2.00	736.14	4.38	0.01
**BCI:Marker**	155.03	77.51	2.00	735.97	1.19	0.30
**Size:Marker**	1896.42	474.10	4.00	735.98	7.30	9.04e-06
**Group:Marker**	1360.28	680.14	2.00	736.02	10.48	3.26e-05
**BCI:Size:Group**	60.70	30.35	2.00	736.11	0.47	0.63
**BCI:Size:Marker**	83.02	20.76	4.00	735.97	0.32	0.86
**BCI:Group:Marker**	306.17	153.08	2.00	735.97	2.36	0.10
**Size:Group:Marker**	544.95	136.24	4.00	735.98	2.10	0.08
**BCI:Size:Group:Marker**	132.26	33.06	4.00	735.97	0.51	0.73

B) Backwards perturbations:

	**Sum Sq**	**Mean Sq**	**NumDF**	**DenDF**	**F value**	**Pr(>F)**
**BCI**	52.82	52.82	1.00	744.86	0.71	0.40
**Size**	13628.75	6814.37	2.00	741.39	91.16	4.00e-36
**Group**	167.94	167.94	1.00	16.87	2.25	0.15
**Marker**	1907.54	953.77	2.00	742.26	12.76	3.56e-06
**Age**	98.01	98.01	1.00	17.06	1.31	0.27
**Trial_order**	1598.70	1598.70	1.00	745.28	21.39	4.42e-06
**Sex**	35.78	35.78	1.00	16.91	0.48	0.50
**Height**	227.44	227.44	1.00	17.36	3.04	0.10
**BCI:Size**	67.08	33.54	2.00	741.62	0.45	0.64
**BCI:Group**	156.41	156.41	1.00	744.36	2.09	0.15
**Size:Group**	55.31	27.66	2.00	741.39	0.37	0.69
**BCI:Marker**	794.43	397.22	2.00	741.35	5.31	0.01
**Size:Marker**	796.76	199.19	4.00	741.27	2.66	0.03
**Group:Marker**	1653.78	826.89	2.00	742.26	11.06	1.85e-05
**BCI:Size:Group**	157.66	78.83	2.00	741.61	1.05	0.35
**BCI:Size:Marker**	158.79	39.70	4.00	741.27	0.53	0.71
**BCI:Group:Marker**	189.36	94.68	2.00	741.35	1.27	0.28
**Size:Group:Marker**	170.51	42.63	4.00	741.27	0.57	0.68
**BCI:Size:Group:Marker**	136.17	34.04	4.00	741.27	0.46	0.77

C) Left perturbations:

	**Sum Sq**	**Mean Sq**	**NumDF**	**DenDF**	**F value**	**Pr(>F)**
**BCI**	31.35	31.35	1.00	751.89	1.48	0.22
**Size**	909.20	454.60	2.00	747.85	21.52	8.15e-10
**Group**	69.55	69.55	1.00	17.87	3.29	0.09
**Marker**	302.18	151.09	2.00	747.40	7.15	8.36e-04
**Age**	50.86	50.86	1.00	17.90	2.41	0.14

**Trial_order**	30.94	30.94	1.00	756.83	1.47	0.23
**Sex**	20.51	20.51	1.00	17.82	0.97	0.34
**Height**	0.58	0.58	1.00	18.08	0.03	0.87
**BCI:Size**	38.23	19.12	2.00	748.24	0.91	0.40
**BCI:Group**	0.13	0.13	1.00	749.44	0.01	0.94
**Size:Group**	88.40	44.20	2.00	747.84	2.09	0.12
**BCI:Marker**	29.28	14.64	2.00	747.17	0.69	0.50
**Size:Marker**	88.74	22.18	4.00	747.10	1.05	0.38
**Group:Marker**	5.40	2.70	2.00	747.40	0.13	0.88
**BCI:Size:Group**	54.53	27.27	2.00	748.08	1.29	0.28
**BCI:Size:Marker**	5.89	1.47	4.00	747.15	0.07	0.99
**BCI:Group:Marker**	12.49	6.25	2.00	747.17	0.30	0.74
**Size:Group:Marker**	147.80	36.95	4.00	747.10	1.75	0.14
**BCI:Size:Group:Marker**	36.76	9.19	4.00	747.15	0.44	0.78

D) Right perturbations:

	Sum Sq	Mean Sq	NumDF	DenDF	F value	Pr(>F)
**BCI**	0.76	0.76	1.00	759.89	0.05	0.82
**Size**	783.89	391.94	2.00	756.64	27.14	4.14e-12
**Group**	6.99	6.99	1.00	18.05	0.48	0.50
**Marker**	39.82	19.91	2.00	756.30	1.38	0.25
**Age**	12.15	12.15	1.00	18.11	0.84	0.37
**Trial_order**	69.88	69.88	1.00	761.33	4.84	0.03
**Sex**	39.55	39.55	1.00	18.05	2.74	0.12
**Height**	114.99	114.99	1.00	18.18	7.96	0.01
**BCI:Size**	94.01	47.00	2.00	757.05	3.25	0.04
**BCI:Group**	114.23	114.23	1.00	758.65	7.91	0.01
**Size:Group**	48.25	24.12	2.00	756.90	1.67	0.19
**BCI:Marker**	1.53	0.77	2.00	756.25	0.05	0.95
**Size:Marker**	66.72	16.68	4.00	756.26	1.15	0.33
**Group:Marker**	8.68	4.34	2.00	756.29	0.30	0.74
**BCI:Size:Group**	136.22	68.11	2.00	756.89	4.72	0.01
**BCI:Size:Marker**	31.23	7.81	4.00	756.28	0.54	0.71
**BCI:Group:Marker**	27.63	13.81	2.00	756.26	0.96	0.38
**Size:Group:Marker**	45.86	11.47	4.00	756.26	0.79	0.53
**BCI:Size:Group:Marker**	79.39	19.85	4.00	756.28	1.37	0.24

Note: Model: value~BCI*Size*Group*Marker + Age + Trial_order + Sex + Height + (1|`Study.code`). ANOVA using Satterthwaite's method. Degrees-of-freedom adjusted for multiple comparisons using Kenward-Roger method.
